# Tumor microenvironment‐responsive delivery nanosystems reverse immunosuppression for enhanced CO gas/immunotherapy

**DOI:** 10.1002/EXP.20220140

**Published:** 2023-07-27

**Authors:** Beibei Chen, Kangli Guo, Xiaoyi Zhao, Zhiwen Liu, Chen Xu, Nana Zhao, Fu‐Jian Xu

**Affiliations:** ^1^ State Key Laboratory of Chemical Resource Engineering Beijing University of Chemical Technology Beijing China; ^2^ Key Laboratory of Biomedical Materials of Natural Macromolecules (Beijing University of Chemical Technology), Beijing Laboratory of Biomedical Materials Beijing University of Chemical Technology Beijing China; ^3^ College of Materials Sciences and Engineering Beijing University of Chemical Technology Beijing China

**Keywords:** CO therapy, hypoxia alleviation, rough surface, tumor microenvironment

## Abstract

Carbon monoxide (CO) gas therapy demonstrates great potential to induce cancer cell apoptosis and antitumor immune responses, which exhibits tremendous potential in cancer treatment. However, the therapeutic efficacy of CO therapy is inhibited by the immunosuppressive tumor microenvironment (TME). Herein, a facile strategy is proposed to construct hollow‐structured rough nanoplatforms to boost antitumor immunity and simultaneously reverse immunosuppression by exploring intrinsic immunomodulatory properties and morphological optimization of nanomaterials. The TME‐responsive delivery nanosystems (M‐RMH) are developed by encapsulating the CO prodrug within hollow rough MnO_2_ nanoparticles and the subsequent surface functionalization with hyaluronic acid (HA). Rough surfaces are designed to facilitate the intrinsic properties of HA‐functionalized MnO_2_ nanoparticles (RMH) to induce dendritic cell maturation and M1 macrophage polarization by STING pathway activation and hypoxia alleviation through enhanced cellular uptake. After TME‐responsive degradation of RMH, controlled release of CO is triggered at the tumor site for CO therapy to activate antitumor immunity. More importantly, RMH could modulate immunosuppressive TME by hypoxia alleviation. After the combination with aPD‐L1‐mediated checkpoint blockade therapy, robust antitumor immune responses are found to inhibit both primary and distant tumors. This work provides a facile strategy to construct superior delivery nanosystems for enhanced CO/immunotherapy through efficient activation of antitumor immune responses and reversal of immunosuppression.

## Graphical abstract

TME‐responsive delivery nanosystems with rough surfaces (M‐RMH) are proposed to enhance CO gas/immunotherapy through immune activation and immunosuppression regulation simultaneously. After the combination with aPD‐L1‐mediated checkpoint blockade therapy, robust antitumor immune responses are found to inhibit both primary and distant tumors.

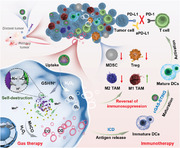

## 1 INTRODUCTION

As an emerging tumor treatment modality, gas therapy has attracted intense interests while versatile delivery nanosystems have been constructed for efficient and controllable delivery of therapeutic gaseous molecules.^[^ ^1^, ^2^] As a green treatment strategy, carbon monoxide (CO) gas therapy demonstrated great potential to induce cancer cell apoptosis through the dysfunction of mitochondria.^[^ ^3^, ^4^ ^]^ However, the direct use of CO (250 parts per million (ppm) for 1 h per day) as a therapeutic molecule inevitably faces the risk of systemic toxicity due to its inherent strong affinity for hemoglobin and low bioavailability.^[^ ^5^, ^6^ ^]^ In recent years, various nanocarriers have been developed for the delivery of CO prodrugs or CO‐releasing molecules to decrease side effects.^[^ ^7^, ^8^, ^9^, ^10^] In addition, CO could induce effective immunogenic cell death (ICD) to increase infiltration of immune cells and effectively activate immune responses.^[^ ^11^, ^12^, ^13^, ^14^ ^]^ However, the current CO gas therapy‐based strategies mainly focus on cytotoxic T lymphocytes (CTLs) activation, while the immune escape and low response rates caused by immunosuppressive tumor microenvironment (TME) are ignored. In particular, representative immunosuppressive cells such as myeloid‐derived suppressor cells (MDSCs), M2 tumor‐associated macrophages (M2‐TAMs) and regulatory T cells (Tregs) play an important role in the inhibition of antitumor immunity.^[^ ^15^, ^16^, ^17^, ^18^, ^19^ ^]^ Among them, M2 macrophages could secrete immunosuppressive cytokines and growth factors which could inhibit T cell proliferation and activation, participate in tumor angiogenesis, and facilitate tumor invasion and metastasis.^[^ ^20^ ^]^ Tregs are considered to inhibit the activation and expansion of tumor antigen‐specific effector T cells through various mechanisms, while MDSCs can suppress effector T cells, NK cells and expand Tregs, finally inhibiting the immune function in TME.^[^ ^15^, ^21^ ^]^ Moreover, immunosuppressive molecules programmed death‐ligand 1 (PD‐L1) overexpressed on tumor cells leads to T cell exhaustion by binding to programmed death receptor‐1 (PD‐1) on T cells.^[^ ^22^, ^23^, ^24^ ^]^ Therefore, it would be desirable to construct nanocarriers for CO gas/immunotherapy to activate immune responses and simultaneously reverse the immunosuppression.

Manganese oxide (MnO_2_) nanomaterials, especially hollow MnO_2_ nanoparticles, have attracted substantial attention due to their high cargo‐loading capacity, TME‐responsive drug release property, and important roles in cancer immunotherapy.^[^ ^25^, ^26^, ^27^ ^]^ MnO_2_ can trigger the decomposition of overexpressed hydrogen peroxide (H_2_O_2_) into oxygen within the TME, which greatly relieve tumor hypoxia and down‐regulate the expression of hypoxia‐inducible factor‐1*α* (HIF‐1*α*),^[^ ^28^, ^29^ ^]^ which may reprogram immunosuppression by downregulating the proportion of Tregs and M2‐TAMs.^[^ ^30^, ^31^ ^]^ In addition, Mn^2+^ released after the responsive degradation of MnO_2_ further induces dendritic cells (DCs) maturation through amplifying the stimulator of interferon genes (STING) activation.^[^ ^32^ ^]^ Meanwhile, it has been found that nanomaterials with rough surfaces can promote cellular uptake, which affects their interaction with both tumor cells and immune cells.^[^ ^33^, ^34^, ^35^, ^36^ ^]^ Therefore, the development of hollow MnO_2_ nanocarriers with rough surfaces will be promising for the combination of CO‐triggered antitumor immunity and reversal of immunosuppressive TME with improved therapeutic efficacy.

**FIGURE 1:**
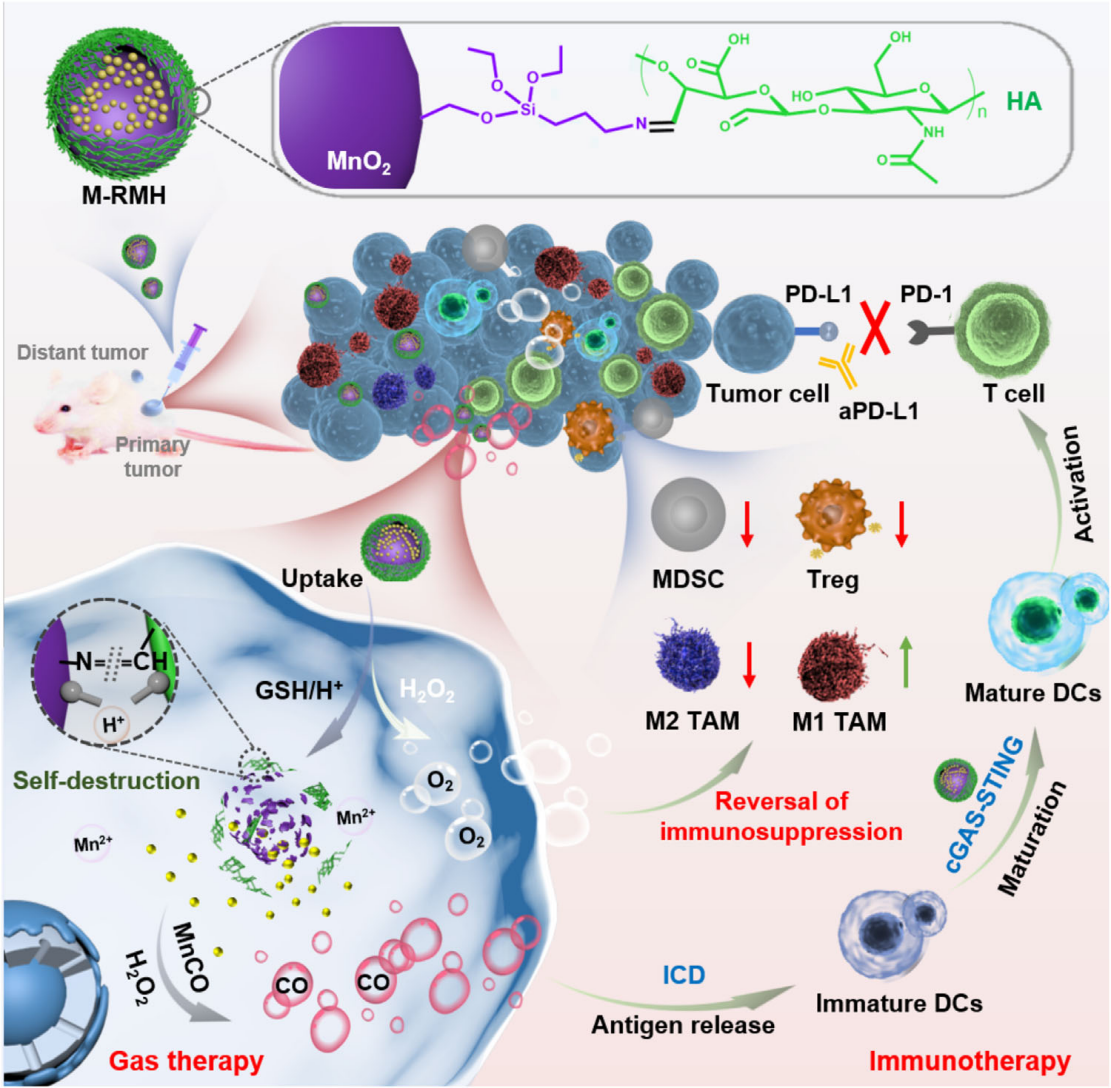
Schematic illustration of TME‐responsive M‐RMH to activate immune responses induced by gas therapy and reverse immunosuppression for complementary gas/immunotherapy.

Herein, we propose TME‐responsive delivery nanosystems with rough surfaces (M‐RMH) to enhance CO gas/immunotherapy through immune activation and immunosuppression regulation simultaneously (Figure 1). RMH was constructed by surface functionalization of hollow rough MnO_2_ nanoparticles with hyaluronic acid (HA), which could deliver CO prodrug (manganese carbonyl, abbreviated as MnCO) into tumor cells and achieve controllable CO release. The resulting M‐RMH nanoparticles could degrade by the reaction with glutathione (GSH) within the acidic TME. Subsequently, the CO release could be triggered in the presence of H_2_O_2_ to induce ICD of tumor cells. Meanwhile, the TME‐responsive production of Mn^2+^ further promotes DC maturation and enhances antitumor immune responses. Moreover, M‐RMH nanoparticles are expected to relieve tumor hypoxia through the reaction with endogenous H_2_O_2_ to modulate immunosuppressive TME. The feasibility of M‐RMH for effective CO gas/immunotherapy was investigated in vitro and in vivo.

## 2 RESULTS AND DISCUSSION

### 2.1 Synthesis and characterization of hollow rough RMH nanoparticles

**FIGURE 2:**
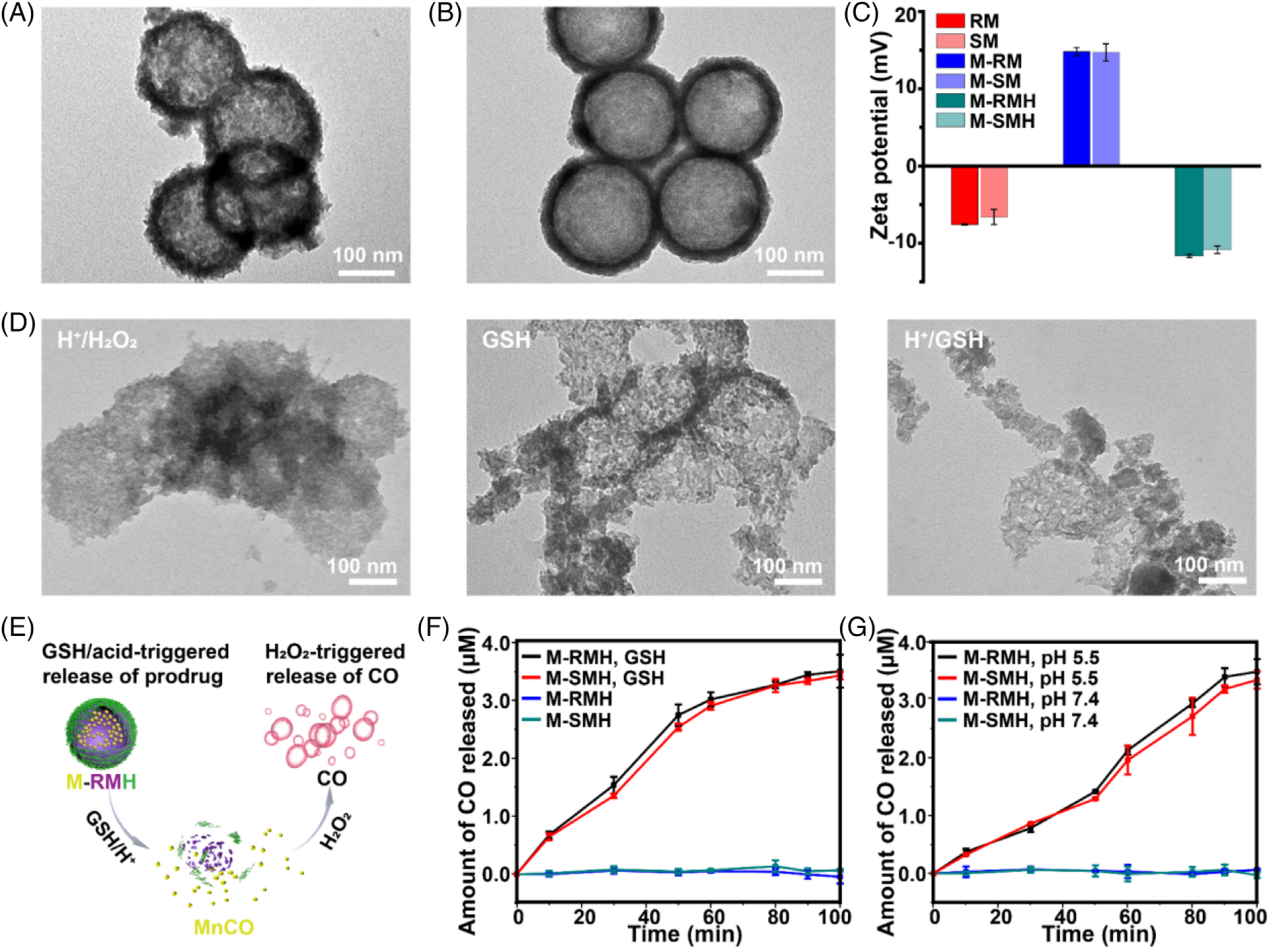
TEM images of (A) RM and (B) SM. (C) Zeta potentials of different nanoparticles (Mean ± SD, *n* = 3). (D) TEM images of M‐RMH after various treatments. (E) Schematic illustration of nanoparticle‐mediated CO release. CO release profiles from M‐RMH or M‐SMH in buffer solutions containing H_2_O_2_ (F) with or without GSH, and (G) with different pH values.

Hollow rough RMH nanoparticles were prepared through the synthesis of hollow rough MnO_2_ nanoparticles (RM) and the subsequent functionalization with HA (Figure 1). Monodispersed SiO_2_ nanoparticles with an average diameter of ≈128 nm (Figure S1A) were first synthesized by a classical Stöber method.^[^ ^27^ ^]^ RM nanoparticles were then prepared by coating SiO_2_ with polydopamine layer, in situ reaction with KMnO_4_, and selective etching of the internal SiO_2_@PDA with Na_2_CO_3_ (Figure S1C,D). Hollow‐structured smooth MnO_2_ nanoparticles (SM, Figure S1B) were also obtained employing SiO_2_ nanoparticles as templates.^[^ ^37^ ^]^ As shown in Figure 2A,B, transmission electron microscope (TEM) images clearly display the hollow feature of rough RM nanoparticles and smooth SM nanoparticles with an average diameter of ≈170 nm.

The hollow structure of MnO_2_ nanoparticles makes them suitable for the loading of CO prodrug MnCO. The MnCO loading efficiency of RM and SM was calculated to be ≈52.8% and ≈50.6%, respectively (Figure S2). Aldehyde‐functionalized HA (HA‐CHO) was synthesized through the oxidation reaction with an absorption peak at 1730 cm^−1^ in the Fourier transform infrared (FTIR) spectra (Figure S3). Furthermore, the oxidation degree of HA‐CHO was determined as ≈44% by the hydroxylamine hydrochloride titration method. After MnCO was loaded in amino‐functionalized RM and SM to produce M‐RM and M‐SM, respectively, HA‐CHO was conjugated onto the surface of nanoparticles to produce M‐RMH and M‐SMH with improved stability and biocompatibility. As shown in Figure 2C and Figure S4, the stepwise change in zeta potential and the increase in hydrodynamic size confirms the successful functionalization of HA on RM, while the zeta potential and hydrodynamic size of M‐SMH and M‐RMH are comparable. Meanwhile, the constant hydrodynamic size of M‐RMH in medium with 10% fetal bovine serum confirmed the feasibility of M‐RMH for in vivo applications (Figure S5). As shown in the TEM images (Figure S6), monodisperse M‐RMH and M‐SMH nanoparticles were obtained. Furthermore, the UV–vis absorption spectra of both M‐SMH and M‐RMH show characteristic peaks of MnCO at 340 nm (Figure S7), indicating successful encapsulation of MnCO.

Since MnO_2_ can be degraded in the TME in the presence of H^+^/H_2_O_2_ and/or GSH,^[^ ^38^ ^]^ the responsive degradation behavior of M‐RMH and M‐SMH was evaluated. As displayed in Figure 2D and Figure S8, the structure of M‐RMH and M‐SMH was destroyed to some extent in the presence of H^+^/H_2_O_2_ or GSH while only fragments could be found after the incubation in the buffer at pH 5.5 in the presence of GSH, verifying the TME‐responsive degradation behavior and enhanced decomposition of MnO_2_ in the acidic TME in the presence of GSH.^[^ ^39^, ^40^ ^]^ Subsequently, the Mn^2+^ release profiles of RMH in different conditions were explored by inductively coupled plasma mass spectrometry (ICP‐MS). As shown in Figure S9, accelerated release of Mn^2+^ in the buffer at pH 5.5 with GSH confirmed the TME‐responsive degradation of RMH. It has been reported that over‐secreted H_2_O_2_ in the TME decomposes into ·OH radicals under the catalysis of MnCO via a Fenton‐like reaction. The resultant ·OH radicals could further oxidize and competitively coordinate with the Mn center, leading to the release of CO.^[^ ^41^ ^]^ Therefore, CO was considered to be released from MnCO triggered by H_2_O_2_ in the TME,^[^ ^42^ ^]^ which could be detected by UV–vis absorption spectroscopy employing hemoglobin as a probe.^[^ ^9^ ^]^ TME‐responsive CO release from M‐RMH and M‐SMH triggered by H_2_O_2_ was then investigated (Figure 2E). As shown in Figure S10, in the presence of GSH or H^+^, the UV‐vis absorbance of hemoglobin at 430 nm decreased with time, whereas the absorbance of carboxyhemoglobin at 410 nm increased continuously due to the continuous generation of CO. The release profiles of CO from M‐RMH and M‐SMH after different treatments reveal that CO could only be released after GSH was added (Figure 2F) or in buffer at pH 5.5 (Figure 2G), confirming the TME‐responsive CO production mediated by M‐RMH and M‐SMH nanoparticles. In addition, similar release behaviors of CO from M‐RM and M‐SM (Figure S11) indicate that HA‐CHO on the surface of nanoparticles didn't affect the responsive release of CO. In addition, the oxygen production ability of MnO_2_ was investigated. As shown in Figure S12, the profiles of the oxygen concentration indicate the efficient generation of O_2_ by M‐RMH, and the amount of oxygen generated under acidic conditions was higher than that under neutral conditions.

### 2.2 Intrinsic immunomodulatory properties of nanoparticles

**FIGURE 3:**
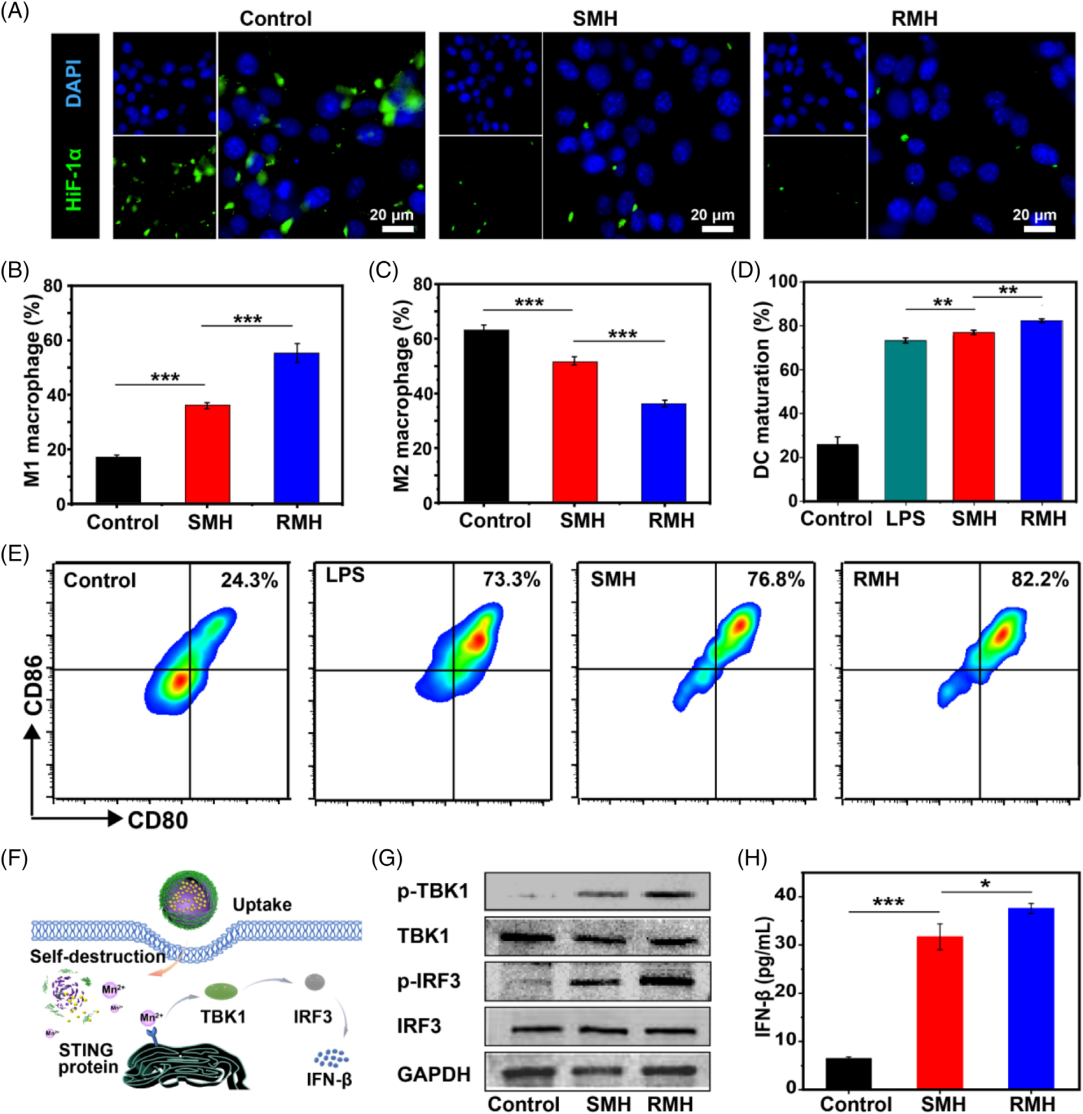
Immunomodulatory effects of RMH and SMH by alleviating hypoxia. (A) Immunofluorescence analysis of HIF‐1*α* expression of 4T1 cells after different treatments. Flow cytometric analysis of (B) M1 macrophages (CD11b^+^CD86^+^) and (C) M2 macrophages (CD11b^+^CD206^+^) after incubation with RM and SM (Mean ± SD, *n* = 3). (D) Flow cytometric analysis and (E) quantification of CD80 and CD86 expression on BMDCs (gated on CD11c^+^ DCs) after different treatments (Mean ± SD, *n* = 3). (F) Schematic illustration of nanoparticles‐mediated activation of the STING pathway. (G) Western blot assay of p‐TBK1, TBK1, p‐IRF3, and IRF3 expression in the BMDCs after different treatments. (H) Secretion of IFN‐*β* secreted in BMDC suspensions after the incubation with SMH and RMH (Mean ± SD, *n* = 3). *p < 0.05, **p < 0.01, ***p < 0.001, analyzed by one‐way ANOVA with Tukey's test.

Since MnO_2_ nanoparticles are supposed to reprogram macrophages toward the M1 phenotype through hypoxia alleviation,^[^ ^27^, ^31^, ^43^ ^]^ the ability of RMH and SMH to alleviate tumor hypoxia was first evaluated. As exhibited in Figure 3A, SMH and RMH downregulated the expression of HIF‐1*α* in 4T1 cells, indicating the capability of SMH and RMH to generate O_2_ within the TME. Notably, RMH resulted in slightly more obvious downregulation of HIF‐1*α* expression, which might be attributed to the enhanced cellular uptake by rough surface of RMH. These results indicate that RMH nanoparticles are promising in the reversal of immunosuppression through hypoxia normalization. In addition, the released Mn^2+^ could also promote macrophage M1‐polarization by activating and sensitizing the cGAS‐STING pathway.^[^ ^44^ ^]^ To further investigate the effect of nanoparticles on macrophage polarization, RMH or SMH were incubated with interleukin 4 (IL‐4)‐pretreated RAW264.7 macrophages (M2 macrophages). As shown in Figure 3B,C and Figure S13, compared with the control group, SMH nanoparticles resulted in an obvious increase in M1 macrophages and decrease in M2 macrophages. Notably, a more significant increase of M1 macrophages was observed after the treatment with RMH, demonstrating the excellent immunomodulatory effect of RMH in macrophage polarization.

As antigen presenting cells, DCs play key roles in antigen presentation and stimulation of antigen‐specific immunity.^[^ ^45^, ^46^ ^]^ Emerging evidence has indicated MnO_2_ could stimulate DC maturation through the activation of the STING pathway.^[^ ^32^, ^47^ ^]^ To investigate the immunoadjuvant property of RMH and SMH, bone marrow‐derived dendritic cells (BMDCs) were incubated with lipopolysaccharide (LPS, positive control), SMH and RMH, respectively. As shown in Figure 3D,E, the matured DCs with evidently upregulated expression of co‐stimulatory molecules CD80 and CD86 were demonstrated after the incubation with SMH and RMH. In addition, RMH were found to significantly increase the maturation of DCs compared with SMH, which might be attributed to the rough surface‐enhanced cellular uptake. To elucidate the mechanisms of DC maturation induced by RMH and SMH, the activation of STING pathway was then investigated (Figure 3F). As shown in Figure 3G, Western Blot analysis demonstrated that the expression of the phosphorylated tank‐binding kinase 1 (p‐TBK1) and phosphorylated interferon regulatory factor 3 (p‐IRF3) were upregulated, which are downstream proteins of the STING pathway. Compared with SMH, RMH induced more obvious upregulation of the p‐TBK1 and p‐IRF3 expression levels. Moreover, the secretion of type I interferon‐*β* (IFN‐*β*) was promoted (Figure 3H), confirming the strong STING activation elicited by RMH. Taken together, the intrinsic immunomodulatory properties of RMH nanoparticles hold great potential in the reversal of immunosuppression and activation of antitumor immunity through downregulation of HIF‐1*α* expression, macrophage repolarization, and DC maturation.

### 2.3 ICD elicited by M‐RMH‐mediated CO therapy

**FIGURE 4:**
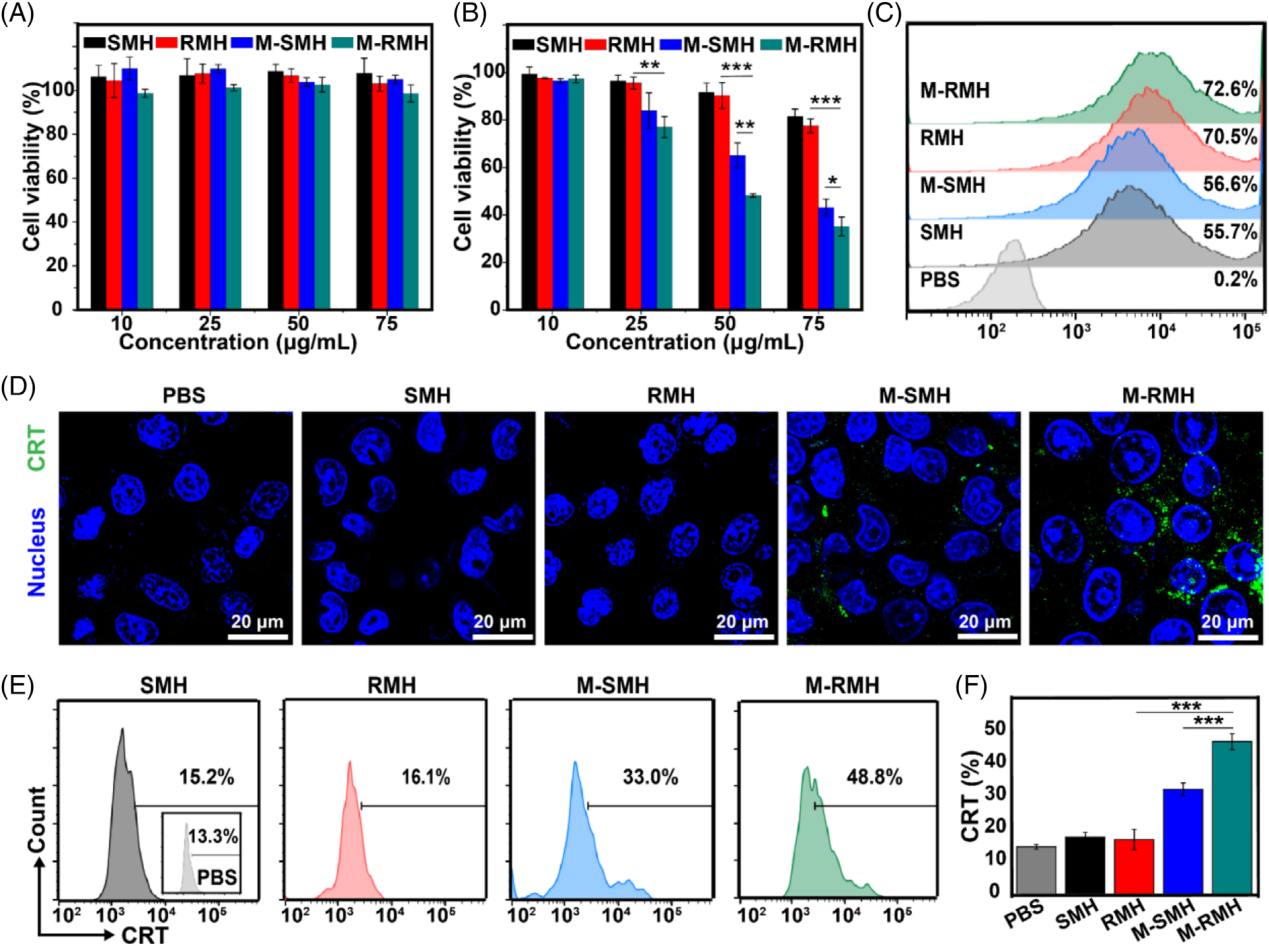
Cell viability of (A) HEK293 cells and (B) 4T1 cells after different treatments (Mean ± SD, *n* = 4). (C) Flow cytometric analysis of 4T1 cells treated with FITC‐labeled SMH, RMH, M‐SMH, and M‐RMH for 4 h. (D) CLSM images of CRT expression on the surface of 4T1 cells after different treatments. (E) Flow cytometric analysis and (F) quantification of CRT expression on the surface of 4T1 cells after various treatments (Mean ± SD, *n* = 3). *p < 0.05, **p < 0.01, ***p < 0.001, analyzed by one‐way ANOVA with Tukey's test.

Enlighted by the efficient CO generation of M‐RMH, ICD of tumor cells triggered by CO therapy was then investigated. As displayed in Figure 4A, the viability of HEK293 cells treated with RMH, SMH, M‐RMH, and M‐SMH was all above 80%, indicating good biocompatibility of nanoparticles. In contrast, M‐RMH, and M‐SMH demonstrated evident cytotoxicity to 4T1 cells compared with RMH and SMH (Figure 4B), owing to the TME and high level of H_2_O_2_ in tumor cells which could trigger responsive degradation of nanoparticles for CO generation. Meanwhile, significantly higher cytotoxicity of M‐RMH against 4T1 cells was found than that of M‐SMH, probably resulting from the enhanced cellular uptake mediated by rough surface.^[^ ^34^ ^]^ To verify this speculation, the cellular uptake of RMH, SMH, M‐RMH, and M‐SMH by 4T1 cells was assessed. The FITC‐labeled nanoparticles were co‐cultured with 4T1 cells for 4 h and then analyzed by flow cytometry. As shown in Figure 4C, RMH and M‐RMH nanoparticles demonstrated much higher internalization ratios (70.5% and 72.6%, respectively) than those of SMH (55.7%) and M‐SMH (56.6%), validating rough surface‐enhanced cellular uptake. In addition, the uptake of M‐RMH by macrophages was investigated by confocal laser scanning microscopy (CLSM). The increased green fluorescence from M‐RMH was found after the incubation with RAW264.7 cells (Figure S14), demonstrating the time‐dependent uptake of M‐RMH by macrophages.

As an “eat‐me” signal, calreticulin (CRT) could be translocated to the surface of cancer cells during ICD and substantially improve the recognition of immune cells for cancer cells.^[^ ^14^ ^]^ To investigate ICD triggered by M‐RMH and M‐SMH, the CRT expression on the surface of 4T1 cell after different treatments was evaluated by CLSM and flow cytometry. As shown in Figure 4D, M‐SMH induced a small fraction of CRT expression on the surface of 4T1 cells while negligible CRT expression could be observed in the PBS, SMH, and RMH groups. Notably, evident CRT exposure on the cell surface could be found in the M‐RMH group, implying enhanced ICD induced by rough surface‐amplified CO therapy. Flow cytometry further confirmed that M‐RMH induced substantially high expression of CRT than that of other groups (Figure 4E,F). Meanwhile, the release of high‐mobility group box 1 (HMGB1) and adenosine triphosphate (ATP) in tumor cells was also evaluated to verify the CO induced ICD. As shown in Figure S15, 4T1 cells treated with M‐RMH and M‐SMH showed higher HMGB1 release and ATP secretion compared with the RMH and SMH groups, verifying ICD induced by CO therapy. In addition, significantly enhanced HMGB1 release and ATP secretion mediated by the M‐RMH group confirmed the advantage of rough surfaces. Collectively, these results indicate that the intrinsic properties of RMH give them promising potential in enhancing CO therapy and triggering immune responses.

To investigate DC maturation induced by the released tumor‐associated antigens during the process of ICD, BMDCs were incubated with the supernatant of 4T1 cells after different treatments. As shown in Figure S16, M‐RMH‐treated cancer cells induced obvious DC maturation, which is significantly higher than the M‐SMH group. This result is consistent with enhanced ICD induced by rough M‐RMH‐mediated CO therapy.

### 2.4 Antitumor efficacy induced by M‐RMH based CO therapy in vivo

**FIGURE 5:**
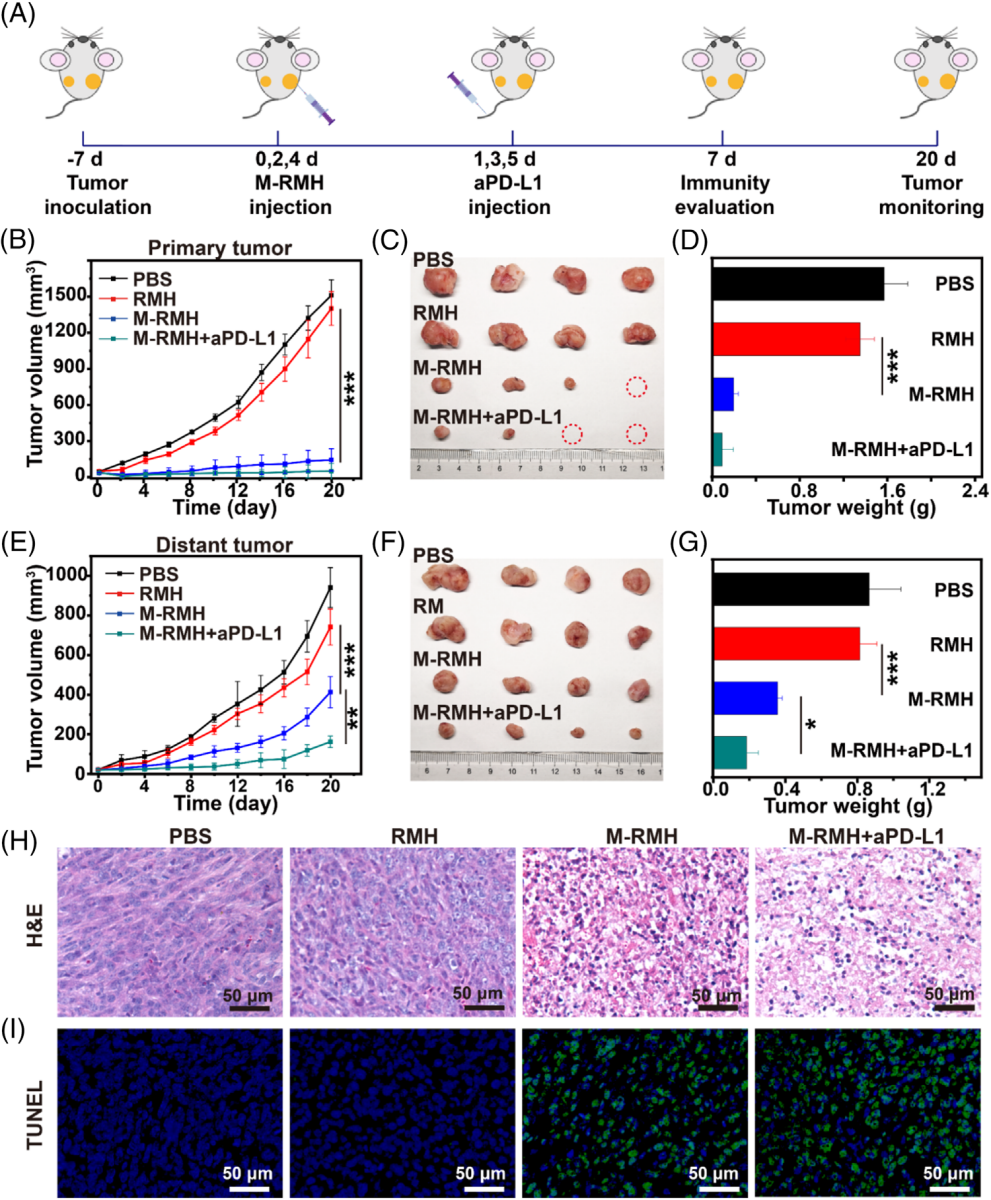
In vivo antitumor efficacy of M‐RMH on a bilateral tumor‐bearing mice model. (A) Schematic illustration of experimental design in vivo. (B) Tumor growth curves, (C) representative photographs, and (D) average tumor weights of primary tumors in mice after the treatment with PBS, RMH, M‐RMH, and M‐RMH + aPDL1, respectively (Mean ± SD, *n* = 4). (E) Tumor growth curves, (F) representative photographs, and (G) average tumor weights of distant tumors of mice after different treatments (Mean ± SD, *n* = 4). (H) H&E and (I) TUNEL staining of primary tumors after different treatments. *p < 0.05, **p < 0.01, ***p < 0.001, analyzed by one‐way ANOVA with Tukey's test.

It is expected that the ideal tumor treatment should not only eradicate the primary tumor, but also stimulate the systemic antitumor immunity to inhibit the growth of metastatic tumors. Encouraged by the excellent intrinsic immunomodulatory properties and antitumor effect of M‐RMH in vitro, we subsequently explored the therapeutic efficacy in vivo employing a bilateral breast tumor model. As shown in Figure 5A, 4T1 cells were subcutaneously injected into the right and left flank of Balb/c mice as the primary tumor and the distant tumor, respectively. These tumor‐bearing mice were randomly divided into four groups, including PBS group, RMH group, M‐RMH group, and M‐RMH + aPD‐L1 group. On days 0, 2, and 4, mice were injected with 100 μL of PBS, RMH, and M‐RMH with RMH concentration of 5 mg mL^−1^, respectively. Mice in the M‐RMH + aPD‐L1 group were intravenously injected with aPD‐L1 (20 μg per mouse) on days 1, 3, and 5. The size of both primary and distant tumors in different groups was recorded every other day. As displayed in Figure 5B,E, compared with RMH, M‐RMH significantly inhibited the growth of primary tumors while distant tumors were suppressed to some extent owing to systemic immune responses mediated by TME‐responsive CO therapy. It is noticed that the M‐RMH + aPD‐L1 group can not only inhibit primary tumor growth, but also considerably inhibit the growth of distant tumors. The satisfactory therapeutic efficacy may be attributed to RMH‐mediated immunomodulatory effects, TME‐responsive CO therapy, and ICD‐induced immunotherapy in combination with immune checkpoint blockade. The corresponding photographs and weights of both primary (Figure 5C,D) and distant (Figure 5F,G) tumors from different groups demonstrated a similar trend to the tumor growth curves. Hematoxylin and eosin (H&E) staining and terminal deoxynucleotidyl transferase‐mediated dUTP nick‐end labeling (TUNEL) analysis following different treatments were utilized to further evaluate the antitumor effect. The results confirmed that M‐RMH + aPD‐L1 group induced the most serious cell apoptosis and necrosis (Figure 5H,I). In addition, no distinct body weight loss was found after various treatments (Figure S17). As displayed in Figure S18, no obvious tissue damage or adverse effect were observed in the histological analysis of major organs (heart, liver, spleen, kidney, and lung) after different treatments, indicating the excellent biocompatibility of M‐RMH nanoparticles.

### 2.5 Antitumor immune responses in vivo

**FIGURE 6:**
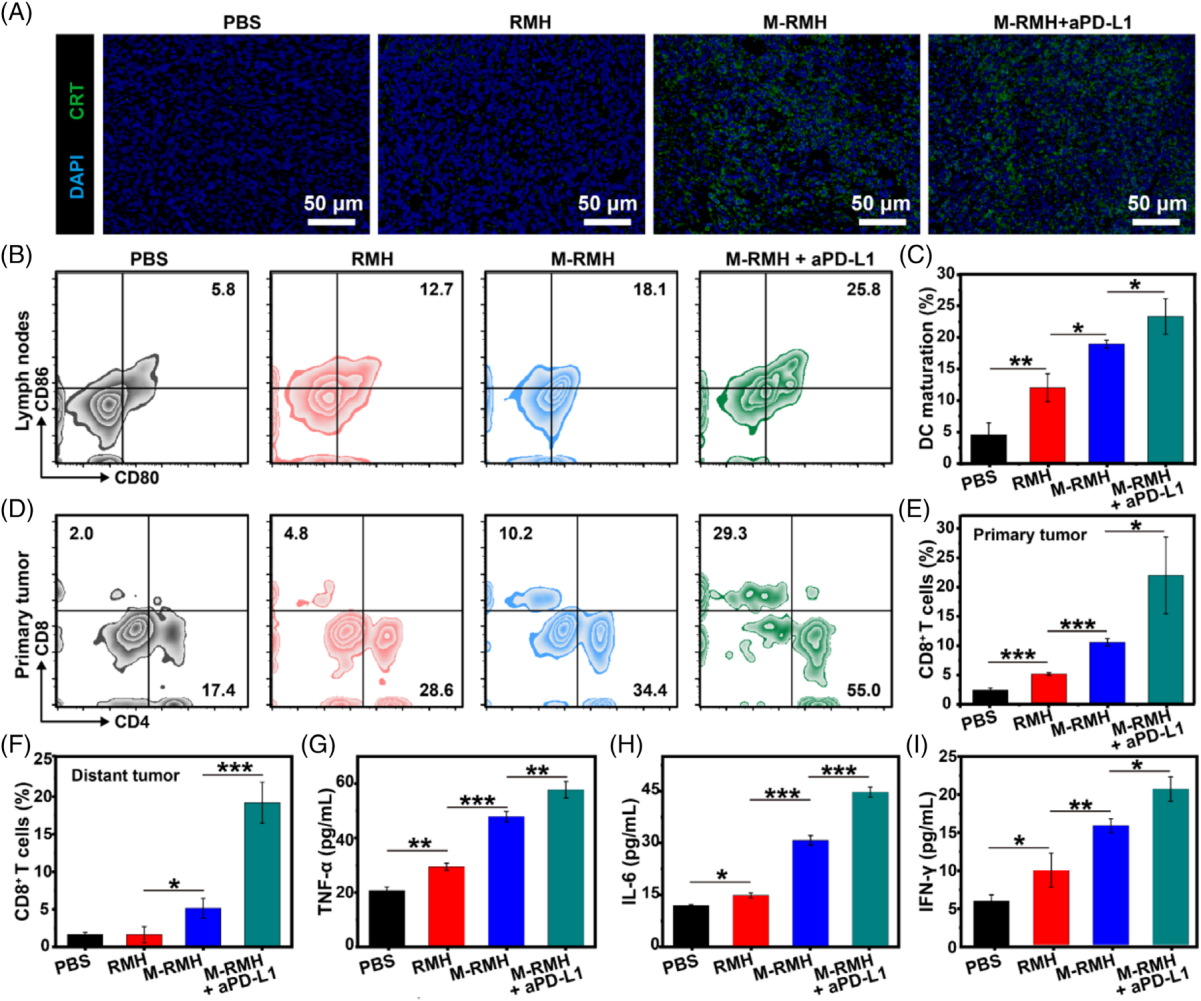
Immune responses elicited by M‐RMH on a bilateral tumor‐bearing mice model. (A) Immunofluorescence images of tumor sections after CRT staining after different treatments. (B) Representative flow cytometry analysis and (C) quantification of matured DC (CD80^+^CD86^+^, gated on CD11c^+^ DCs) in lymph nodes after different treatments (Mean ± SD, *n* = 3). (D) Representative flow cytometry analysis of CD4^+^ and CD8^+^ T cells (gated on CD3^+^ T cells) in the primary tumors after various treatments. (E) Quantification of CD8^+^ T cells in the primary tumors after various treatments (Mean ± SD, *n* = 3). (F) Quantification analysis of CD8^+^ T cells in distant tumors after different treatments (Mean ± SD, *n* = 3). Cytokine levels of (G) TNF‐*α*, (H) IL‐6, and (I) IFN‐*γ* in the serum of mice after various treatments (Mean ± SD, *n* = 3). *p < 0.05, **p < 0.01,***p < 0.001, analyzed by one‐way ANOVA with Tukey's test.

To reveal the mechanism of excellent antitumor effect mediated by M‐RMH, the antitumor immunity on a bilateral 4T1 tumor‐bearing mice model was investigated. As shown in Figure 6A, obvious CRT exposure was found in the tumors treated with M‐RMH and M‐RMH + aPD‐L1 due to ICD induced by TME‐responsive CO therapy. Furthermore, to confirm the antitumor immune responses, immune cells and cytokines of different groups were collected and detected. Since both the generated tumor‐associated antigens induced by CO therapy and intrinsic immunoadjuvant effect of M‐RMH could induce DC maturation, which plays a pivotal role in activating T cell‐mediated immune responses, the percentage of matured DCs in the lymph nodes was evaluated by flow cytometry. As demonstrated in Figure 6B,C, compared with PBS, remarkably higher percentage of matured DCs (CD11c^+^CD80^+^CD86^+^) in the RMH group (≈11%) verified their immunoadjuvant effect through the STING activation. The substantially enhanced DC maturation was further observed in the M‐RMH (≈18%) and M‐RMH + aPD‐L1 groups (≈23%), respectively. Furthermore, the percentages of CD4^+^ helper T cells and CD8^+^ CTLs were investigated to evaluate the activation of antitumor immunity. The infiltration of CD8^+^ CTLs and CD4^+^ T cells was significantly increased in both primary and distant tumors after the treatment with RMH group (Figure 6D–F and Figures S19, S20), confirming the contribution of RMH‐mediated DC maturation and modulation of immunosuppressive TME. In addition, substantially increased percentage of tumor‐infiltration CTLs was found in the M‐RMH group, which might be ascribed to ICD induced by CO therapy. Furthermore, the activation of distal antitumor immunity was verified to suppress the untreated distant tumor growth and prevent tumor metastasis. Meanwhile, the proportion of CTLs in tumors was further enhanced in the combination treatments of M‐RMH with aPD‐L1, indicating the robust antitumor immunity of CO therapy in combination with immune checkpoint blockade. The enhancement of CTLs was much more obvious in distant tumors (Figure 6E,F), which is also consistent with the therapeutic effectiveness. The activation of the T cell‐mediated antitumor immunity was further verified. The same trend of CTLs population in the spleen further confirmed the activation of immune responses mediated by different treatments (Figure S21). Moreover, the levels of tumor necrosis factor‐*α* (TNF‐*α*), interleukin‐6 (IL‐6), and interferon‐*γ* (IFN‐*γ*) cytokines in serum were detected by enzyme‐linked immunosorbent assay (Figure 6G–I). Compared with other groups, the M‐RMH + aPD‐L1 group stimulated the strongest antitumor immune responses. Collectively, M‐RMH in combination with aPD‐L1 was proved to stimulate robust antitumor immunity to achieve superior therapeutic efficacy and antimetastatic effects.

### 2.6 Reversal of immunosuppression

**FIGURE 7:**
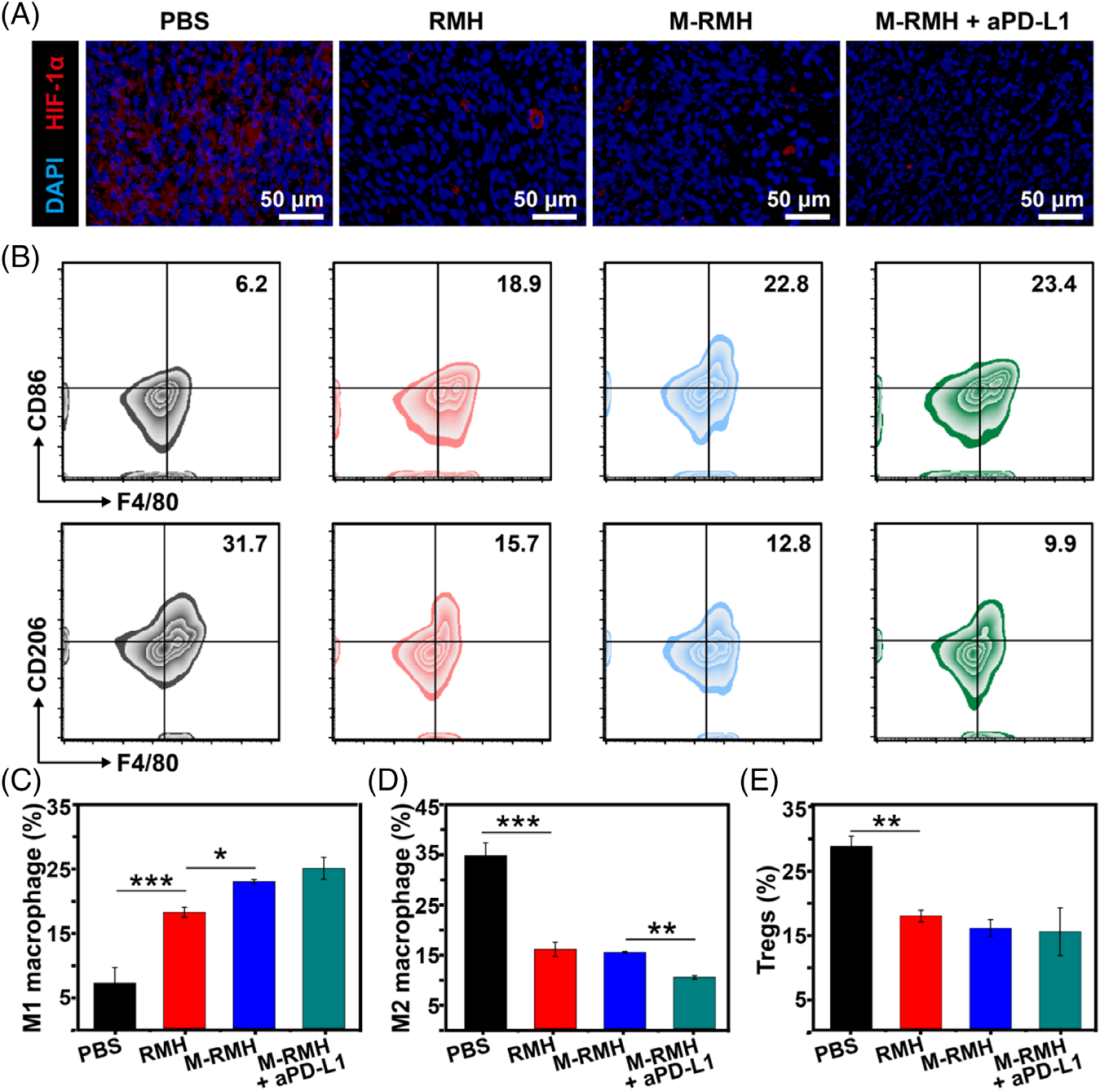
Reversal of immunosuppression on a bilateral tumor‐bearing mice model. (A) Immunofluorescence assay showing HIF‐1*α* expression in primary tumor tissues from mice after different treatments. (B) Representative flow cytometry analysis of M1 (CD11b^+^F4/80^+^CD86^+^) and M2 macrophages (CD11b^+^F4/80^+^CD206^+^) in primary tumors after various treatments. Quantification analysis of (C) M1 (CD11b^+^F4/80^+^CD86^+^) and (D) M2 macrophages (CD11b^+^F4/80^+^CD206^+^) in primary tumors. (E) Quantification of Tregs (CD3^+^CD4^+^Foxp3^+^) in primary tumors after different treatments. *p < 0.05, **p < 0.01, ***p < 0.001, analyzed by one‐way ANOVA with Tukey's test.

Encouraged by the promising results of HIF‐1*α* expression downregulation and macrophages polarization in vitro, the effects of RMH‐mediated treatments on the reversal of immunosuppression were investigated, including macrophage polarization, as well as the modulation of Tregs and MDSCs suppression. As shown in Figure 7A, HIF‐1*α* expression was evidently downregulated after the treatment with RMH, implying the hypoxia attenuation capacity of RMH in vivo. Meanwhile, M‐RMH and M‐RMH + aPD‐L1 treatment showed similar downregulation of HIF‐1*α* expression. Since the ability of RMH to alleviate hypoxia and the generated tumor‐associated antigens induced by CO therapy may also repolarize TAMs to antitumor M1 phenotype, the proportion of TAMs in the primary tumors was studied. As shown in Figure 7B–D, compared with the PBS group, the percentage of M1 macrophages (CD11b^+^F4/80^+^CD86^+^) increased obviously while M2 macrophages (CD11b^+^F4/80^+^CD206^+^) were substantially reduced after the treatment with RMH, implying the ability of RMH to reprogram TAMs by alleviating hypoxia. Furthermore, M‐RMH and M‐RMH + aPD‐L1 demonstrated enhanced M1 macrophages polarization, which could be attributed to ICD of tumor cells induced by CO therapy.^[^ ^34^ ^]^ In addition, other immunosuppressive cells including Tregs (CD3^+^CD4^+^Foxp3^+^) and MDSCs (CD45^+^CD11b^+^Gr‐1^+^) were assessed by flow cytometry. As shown in Figure 7E and Figure S22, the proportion of Tregs in primary tumors treated with RMH was much lower than that in the PBS group, while M‐RMH and M‐RMH + aPD‐L1 groups showed similar results. Furthermore, a considerable reduction of MDSCs was found after RMH‐based treatments (Figure S23). Taken together, the successful reversal of immunosuppression of TAM polarization to M1 macrophages and the downregulation of Tregs and MDSCs was believed to contribute to the robust T cell‐mediated antitumor immunity.

## 3 CONCLUSIONS

In summary, TME‐responsive delivery nanosystems with rough surfaces (M‐RMH) were successfully constructed for enhanced CO gas/immunotherapy. M‐RMH was fabricated by the loading of MnCO in hollow rough MnO_2_ nanoparticles. After TME‐responsive degradation of RMH, controllable CO release was achieved through the reaction of H_2_O_2_ with MnCO, which could induce ICD of tumor cells to activate antitumor immune responses. In addition, the intrinsic immunomodulatory properties of RMH contribute to the immunotherapy. DC maturation was induced through the STING activation, which could boost antitumor immune responses. Moreover, M‐RMH nanoparticles could reverse immunosuppression by M1 macrophage polarization through hypoxia alleviation. It is interesting that RMH nanoparticles with rough surfaces performed much better than the smooth counterparts in ICD induction, DC maturation, and macrophage polarization, which could be attributed to enhanced cellular uptake. Notably, significantly enhanced CD8^+^ CTLs infiltration was mediated by M‐RMH while significantly downregulation of Tregs, M2 macrophages, and MDSCs was observed on a bilateral breast tumor model. In combination with aPD‐L1 antibody, more significant inhibition of distant tumors was observed. The current work provides a new strategy for effective CO gas/immunotherapy by immune activation and immunosuppression regulation simultaneously.

## 4 EXPERIMENTAL SECTION

Experimental details are provided in the Supporting Information.

## CONFLICT OF INTEREST STATEMENT

The authors declare no conflicts of interest.

## Supporting information

Supporting Information

## Data Availability

All data related to this study are present in the article and in the Supporting Information. Any other data associated with this work are available from the corresponding authors upon request.
