# Supplementary material for: Tumor microenvironment‐responsive delivery nanosystems reverse immunosuppression for enhanced CO gas/immunotherapy
Source: Exploration (Beijing). 2023 Jul 27;3(6):20220140. doi: 10.1002/EXP.20220140 (PMC10742199; doi:10.1002/EXP.20220140)
Supplement: Supplementary file 1 — Supporting Information [file EXP2-3-20220140-s001.docx]

**Supporting information**

**Tumor microenvironment-responsive delivery nanosystems reverse immunosuppression for enhanced CO gas/immunotherapy**

*Beibei Chen,^1,2,3^ Kangli Guo,^1,2,3^ Xiaoyi Zhao,^1,2,3^ Zhiwen Liu,^1,2,3^ Chen Xu,^1,2,3^ Nana Zhao, ^1,2,3^* Fu-Jian Xu ^1,2,3^**

^1^State Key Laboratory of Chemical Resource Engineering, Beijing University of Chemical Technology, Beijing, 100029, China

^2^Key Laboratory of Biomedical Materials of Natural Macromolecules (Beijing University of Chemical Technology), Beijing Laboratory of Biomedical Materials, Beijing University of Chemical Technology, Beijing, 100029, China

^3^College of Materials Sciences and Engineering, Beijing University of Chemical Technology, Beijing, 100029, China

* To whom correspondence should be addressed

E-mail addresses: xufj@mail.buct.edu.cn (F. J. Xu); zhaonn@mail.buct.edu.cn (N. Zhao).

**EXPERIMENTAL SECTION**

**Materials**

Ferric chloride (FeCl_3_·6H_2_O, 99%), manganese carbonyl (98%), hemoglobin (M_W_ 65 Da), 2’,7’-dichlorofluorescein-diacetate (DCFH-DA 97%), glutathione (GSH, 98%), and lipopolysaccharide (LPS) were purchased from Sigma-Aldrich (USA). Tetraethoxysilane (TEOS, 98%), tetramethylbenzidine (TMB, 98%), 3-aminopropyl triethoxysilane (APTES), triethylamine (TEA), and 3-hydroxytyramine hydrochloride (98%) were purchased from Energy Chemical (China). Sodium citrate (NaCit, 99%), sodium acetate (NaAc, 99%), sodium hydroxide (NaOH), carbonic acid (Na_2_CO_3_, 99%), potassium permanganate (KMnO_4_), ethanol (>99%), methanol (>99%), ammonium hydroxide (NH_4_OH, 25 wt%), dimethyl sulfoxide (DMSO, >90%), and hydrogen peroxide (H_2_O_2_, 30%) were provided by Beijing Chemical Co. (China). 4,5-Dimethylthiazol-2yl)-2,5-diphenyl tetrazolium bromide (MTT), 4’,6-diamidino-2-phenylindole (DAPI) were purchased from Beijing Solarbio Science & Technology Co., Ltd (China). Murine granulocyte-macrophage colony-stimulating factor (GM-CSF) and murine interleukin 4 (IL-4) were purchased from Peprotech (USA). Antibodies used for flow cytometry were all purchased from eBioscience (USA). Milli-Q Ultrapure water (18.2 MΩ) was used in all experiments.

**Synthesis of SM and RM** **nanoparticles**

Hollow MnO_2_ nanoparticles were synthesized by using SiO_2_ nanoparticles as the templates.^[^^[[1]](#endnote-1)]^ For the synthesis of SiO_2_ nanoparticles, 2 mL of NH_3_·H_2_O were added in 1.5 mL of TEOS, and then the mixture was stirred for 6 h at 30 ^o^C. SiO_2_ nanoparticles were collected by centrifugation (8000 rpm, 10 min) and washed with H_2_O. Then, 30 μL of ethanol and 10 mL of KMnO_4_ (60 mg mL^-1^) was successively added in 10 mL of SiO_2_ nanoparticles (8 mg mL^-1^) and the mixture were stirred for 6 h. The as-prepared SiO_2_@MnO_2_ was dissolved in 1 M Na_2_CO_3_ aqueous solution at 60 ^o^C for 12 h. Hollow SM nanoparticles were obtained by centrifugation and washing with H_2_O.

For the synthesis of RM nanoparticles, dopamine-coated silica (SiO_2_@PDA) nanoparticles were firstly synthesized as templates. 100 mg of SiO_2_ and 160 mg of dopamine hydrochloride were dispersed in Tris buffer (pH 8.5, 10 mM) and stirred at room temperature for 24 h. The resulting SiO_2_@PDA nanoparticles were separated by centrifugation, and then washed with H_2_O. Subsequently, 7 mL of KMnO_4_ (5.3 mg mL^-1^) were dropwise added into 9 mL of SiO_2_@PDA (2.7 mg mL^-1^) solution, and then 0.1 mL of H_2_SO_4_ (1 M) was added under ultrasonication. After 6 h, the precipitate was obtained by centrifugation at 8000 rpm and etched with Na_2_CO_3_ solution (1 M) at 60 ^o^C for 2 h to obtain hollow RM nanoparticles.

**Synthesis of M-RMH and M-SMH**

The aldehyde groups-bearing hyaluronic acid (HA-CHO) was obtained by the reaction of hyaluronic acid and sodium periodate, following the previously reported method.^[^^[[2]](#endnote-2)]^ Briefly, 1 g of hyaluronic acid was dissolved in 100 mL of deionized water, and then 0.532 g of sodium periodate was added. The mixed solution was stirred at room temperature for 24 h. Subsequently, 0.6 mL of ethylene glycol was added. The product was dissolved in water, dialyzed against deionized water, and obtained by lyophilization. The oxidation degree of the HA-CHO was determined by the titration analysis of hydroxylamine hydrochloride.^[^[[3]](#endnote-3)^]^ Then 1 mg of RM was dispersed in 10 mL of ethanol/H_2_O (v/v: 9:1) solution. APTES (1 mL) and TEA (1 mL) were added and the solution was kept stirring for 24 h. Aminated RM (RM-NH_2_) was obtained by centrifugation (8000 rpm, 10 min).

For the preparation of M-RMH, 1 mg of MnCO was dissolved in 1 mL of methanol and then 1 mL of RM-NH_2_ (0.3 mg) in methanol solution was added into the above solution. Afterwards, the mixture was vacuum dried at room temperature. The obtained nanoparticles were collected by centrifugation and washed with methanol and PBS to obtain the M-RM nanoparticles. Finally, the HA-CHO (1 mg) was added into 0.5 mL of M-RM solution and stirred at room temperature for 4 h, and the nanoparticles were obtained by centrifugation. Similarly, M-SMH nanoparticles were also obtained by same preparation procedure.

**Characterization**

The morphology of nanoparticles was observed by transmission electron microscopy (TEM, Hitachi HT-7800, Hitachi, Japan). The particle size and zeta potential of nanoparticles were measured using a Zetasizer Nano ZS (Malvern Instruments, Southborough, MA). Fourier transform infrared spectroscopy analysis (FTIR, Nicolet IS 10, Thermo Scientific, USA) was conducted to determine the functional groups of the nanoparticles. Ultraviolet-visible-near-infrared (UV-vis-NIR) absorption spectra were performed on a SHIMADZU U3600 spectrometer (SHIMADZU, Japan).

**MnCO loading capacity**

The MnCO loading capacity of M-RM and M-SM was analyzed by measuring the UV–vis absorbance at 340 nm. Briefly, the supernatant was collected, and the absorbance at 340 nm was recorded. According to the following equation, the MnCO loading capacity was calculated: Loading capacity (%) = (W-W_1_)/W_2_*100%, where W represents the initial feeding amount of MnCO, W_1_ represents the MnCO content in the supernatant, and W_2_ represents the mass of M-RM or M-SM.

**Degradation of M-RMH and M-SMH**

1 mg mL^-1^ of M-RMH or M-SMH was incubated in of PBS buffers (pH = 5.5 or pH = 7.4) with H_2_O_2_ (100 μM) or GSH (10 mM) for 8 h. The morphology of nanoparticles was monitored by TEM at the different time points.

***In vitro* CO release**

The CO release from M-RMH and M-SMH was measured according to the previously reported method.^[^[[4]](#endnote-4)^]^ The released CO was detected by measuring the conversion of hemoglobin (Hb) to carboxyhemoglobin (HbCO). Firstly, bovine hemoglobin was dissolved in 4 mL of deoxygenated PBS. Then sodium dithionite was added to the solution to reduce the hemoglobin. Afterwards, 200 μL of M-RMH and M-SMH suspension (0.3 mg mL^-1^) was added to the mixed solution, followed by the addition of 1 mL of PBS buffers (pH = 5.5 or pH = 7.4) containing H_2_O_2_ (100 μM) with or without GSH (10 mM) to mimic tumor environment. The UV-vis absorption spectra of M-RMH or M-SMH were recorded every 10 min. The concentration of released CO was calculated as follows: C_co_ = C_Hb_ (528.6 × I_410nm_ -304 × I_430nm_) / (216.5 × I_410nm_ + 442.4 × I_430nm_), where C_CO_ is the concentration of released CO, C_Hb_ is the concentration of bovine hemoglobin, and I_410 nm_ and I_430 nm_ are the absorbance of the mixed solution at the wavelength of 410 and 430 nm, respectively.

***In vitro* O_2_ generation**

The O_2_ generation ability of M-RMH was evaluated by measuring the oxygen production with a dissolved oxygen meter. Specifically, 100 μM H_2_O_2_ was dissolved in 2 mL of PBS buffer solution (pH = 5.5 or pH = 7.4), followed by the addition of 2 mg mL^-1^ M-RMH. The oxygen concentration was immediately detected and the data have been continuously recorded for 10 minutes.

**Cells culture**

4T1, HEK293, and RAW264.7 cell lines were provided by American Type Culture Collection (ATCC, Rockville, MD). 4T1 cells were cultured in Roswell Park Memorial Institute (RPMI) medium, and RAW264.7 and HEK293 cells were cultured in Dulbecco’s modified eagle medium (DMEM), supplemented with 10% heat-inactivated fetal bovine serum (FBS), 100 units mL^-1^ of penicillin, and 100 mg mL^-1^ of streptomycin at 37 ^o^C, under 5% CO_2_ and 95% relative humidity atmosphere.

**Intracellular hypoxia detection**

For the measurement of HIF-1*α* expression, 4T1 cells were seeded onto glass bottom culture dishes and incubated with RM or SM nanoparticles (50 μg mL^-1^). Then the cells were washed and fixed with 4% paraformaldehyde for 10 min at room temperature. The cells were then incubated with anti-HIF-1*α* antibody (proteintech, USA) for 2 h and Alexa Fluor 488-conjugated secondary antibody (Beyotime) for 1 h at room temperature according to manufacturer’s instructions. Thereafter, the cells were stained with DAPI for 5 min and observed using CLSM.

***In vitro* assessments of macrophage polarization**

RAW264.7 cells were seeded into 6-well plates (2 × 10^5^ cells well^-1^) and incubated with IL-4 (20 ng mL^-1^) for 24 h to obtain M2-like macrophage. Then RMH or SMH (50 μg mL^-1^) was incubated with M2 macrophage at 37 °C for 24 h. The cells were harvested for flow cytometry analysis. RAW264.7 cells were washed and then incubated with antibodies (APC-anti-CD86, FITC-anti-CD11b) or antibodies (APC-anti-CD206, FITC-anti-CD11b) at 4 °C, respectively. After 30 min, cells were washed thoroughly and detected with flow cytometry (BD Accuri C6 plus, USA).

***In vitro* BMDC stimulation**

To investigate DC maturation induced by nanoparticles, bone-marrow-derived dendritic cells (BMDCs) were generated from the 7-week-old mice.^[^^[[5]](#endnote-5)]^ BMDCs cells were incubated with 50 μg mL^-1^ SMH or RMH or LPS (1 µgmL^-1^) for 24 h and then stained with anti-CD11c-FITC, anti-CD80-PE and anti-CD86-APC antibodies. DCs maturation was analyzed by flow cytometry.

To investigate the effect of M-RMH-mediated antigen release on DC activation, 4T1 cells were first seeded in 24-well plates at a density of 5×10^4^ cells well^-1^ and incubated with 500 μL of SMH, RMH, M-SMH, and M-RMH (50 μg mL^-1^), repectively for 24 h. Then BMDCs were incubated with the supernatant of 4T1 cells after different treatments for 24 h. The stimulation of BMDCs was examined as mentioned above.

**Western blot**

For western blots of TBK1, p-TBK1, IRF3 and p-IRF3 expressions, BMDCs were seeded in 6-well plates (10^6^ cells well^-1^) and treated with PBS, SMH, and RMH for 24 h, respectively. Subsequently, the cells were washed with PBS and collected for a standard Western blot process.

**Cytotoxicity assay**

The cytotoxicity of nanoparticles was evaluated in HEK293 cell line. Briefly, HEK293 cells were seeded in a 96-well plate at a density of 10^4^ cells per well and cultured for 24 h. The culture media were then replaced by fresh media containing various concentrations of SMH, RMH, M-SMH, and M-RMH, respectively and cultured for 24 h. Finally, the cell viability was measured by typical MTT assay following our previous reports.^[^^[[6]](#endnote-6)]^

***In vitro* CO therapy**

To evaluate the therapeutic effect of M-RMH and M-SMH *in vitro*, 4T1 cells were seeded in a 96-well plate at a density of 10^4^ cells per well and cultured for 24 h. And then the culture media were replaced with 100 μL of fresh media containing various concentrations of SMH, RMH, M-SMH or M-RMH and cultured for 24 h. The cell viability was measured by MTT assay.

**Cellular internalization**

6×10^5^ 4T1 cells well^-1^ were seeded in 6-well plates and cultured for 24 h. Then, the medium was replaced with 2 mL of fresh RPMI medium containing FITC-labele RMH, SMH, M-RMH, and M-SMH nanoparticles (50 μg mL^-1^), respectively. After 4 h, the cells were washed with PBS and the percentage of FITC-positive cells was determined by flow cytometry.

For RAW264.7 cell uptake, 1×10^6^ RAW264.7 cells well^-1^ were seeded in 6-well plates and cultured for 24 h. Then, the medium was replaced with 2 mL of fresh DMEM medium containing FITC-labele M-RMH nanoparticles (50 μg mL^-1^), respectively. After 1-6 h, the cells were washed with PBS and labeled with DAPI for 10 min, and determined by CLSM.

**Characterization of immunologic cell death (ICD) *in vitro***

For immunofluorescence detection of calreticulin (CRT) expression, 4T1 cells were seeded onto glass bottom culture dishes and then incubated with PBS, SMH, RMH, M-SMH or M-RMH (50 μg mL^-1^) for 24 h. The cells were then washed twice with PBS, fixed with 4% paraformaldehyde at room temperature, incubated with the anti-CRT antibody (Beyotime) for 2 h, and then incubated with Alexa Fluor 488-conjugated secondary antibody (Beyotime) for another 1 h. Finally, the cells were stained with DAPI for 5 min and observed using laser scanning confocal microscope (Leica SP8).

For flow cytometric analysis of CRT exposure, 4T1 cells (6 × 10^5^ cells well^-1^) were seeded in a 6-well plate for one day. Then the cells were incubated with PBS, SMH, RMH, M-SMH or M-RMH (50μg mL^-1^) for 24 h. Thereafter, the cells were harvested, washed with PBS, fixed in 0.25% paraformaldehyde for 5 min, and incubated with the anti-CRT antibody for 1 h. After being washed with PBS twice, the cells were incubated with Alexa Fluor 488-conjugated secondary antibody for 1 h. Finally, the cells were washed twice with PBS and collected for flow cytometry. The release of HMGB1 and ATP was examined by the HMGB1 ELISA Kit and Enhanced ATP assay kit, respectively. Briefly, 4T1 cells were seeded in 24-well plates. Then the cells were incubated with PBS, SMH, RMH, M-SMH or M-RMH (50 μg mL^-1^) for 24 h. Then, the supernatant was collected and the release of HMGB1 was detected by the HMGB1 ELISA Kit (Elabscience Biotechnology Co., Ltd) while the release of ATP was detected by an Enhanced ATP assay kit (Beyotime) according to the manufacture’s protocols.

**Antitumor immunity study in a bilateral 4T1 tumor model**

Female BALB/c mice (6 weeks old, weight 16-18 g) were purchased from Beijing Vital River Laboratory Animal Technology Co., LTD (Beijing, China). Animal studies were approved by Ethical Committee of Chinese Academy of Medical Sciences and Peking Union Medical College and performed under legal protocols. To establish the bilateral 4T1 tumor-bearing mouse model, the mice were subcutaneously inoculated with 4T1 cells into the left flank (1 × 10^6^ cells/mouse, primary tumor) and and right (2×10^5^ cells/mouse, distant tumor) flank, respectively. After 7 days, the mice were randomly divided into four groups with four mice in each group, including PBS group, RMH group, M-RMH group, and M-RMH + aPD-L1 group. The size of primary and distant tumors and the body weight of mice were recorded every two days. After twenty days, all the mice were sacrificed and the tumors were weighed, imaged, and dissected, prior to H&E and immunofluorescence analysis.

In addition, another batch of mice were treated aforementioned and sacrificed on the seventh day. Then the primary tumors, distant tumors, spleens, lymph nodes, and peripheral blood were collected for the analysis of macrophages, MDSCs, Tregs, T cells, and DCs by flow cytometry and immunofluorescence analysis of HIF-1*α* and PD-L1. The proinflammatory cytokines including IL-6, TNF-*α*, and IFN-*γ* in serum were tested by ELISA (Dakewe Biotech, China).

**Analysis of antitumor immune responses *in vivo***

Tumors obtained from mice were digested by collagenase IV, hyaluronidase and DNase I (Solarbio, China) to obtain the single-cell suspension and filtered through 75 μm filters. The cell suspensions of primary tumors were divided into quadruplicate and blocked with 2% FBS. For M1 macrophage analysis, the cells were stained with anti-CD11b-FITC, anti-F4/80-PerCP-Cy5.5, and anti-CD86-APC antibodies. For M2 macrophage analysis, the cells were stained wiht anti-CD11b-FITC, anti-F4/80-PerCP-Cy5.5 and anti-CD206-APC antibodies. For T cells activation analysis, the cell suspensions were stained with anti-CD3-FITC, anti-CD4-PE and anti-CD8a-APC antibodies. For Tregs analysis, the cell suspensions were stained with anti-CD3-FITC, anti-CD4-PE, and anti-Foxp3-PE-Cy5.5 antibodies. For MDSCs analysis, the cell suspensions were stained with anti-CD11b-FITC, anti-CD45-PE-Cy7 and anti-Gr-1-PE antibodies. Finally, cells were collected and analyzed by flow cytometry. The population of T cells in distant tumors were also examined by flow cytometry.

On the other side, spleens were ground and dispersed in RPMI medium, then the red blood cells were discarded by red blood cell lysis buffer. Subsequently, splenic cells were stained with anti-CD3-FITC, anti-CD4-PerCP-Cy5.5 and anti-CD8a-APC antibodies after being blocked with 2% FBS. Flow cytometry was used to analyze the splenic cells.

For DC maturation analysis, the inguinal lymph nodes of mice were gathered and filtered to gain single-cell suspension. Then, the cell suspensions were blocked with 2% FBS and stained with anti-CD11c-FITC, anti-CD80-PE and anti-CD86-APC antibodies for flow cytometry assay.

**Statistical analysis**

Data were presented as means ± standard deviation and are from at least three independent experiments. The differences between two groups were calculated by using one-way ANOVA with Tukey’s Test. **P* < 0.05, ***P* < 0.01, or ****P* < 0.001 was considered statistically significant. The statistical analysis were performed using GraphPad GraphPad Prism software.

**
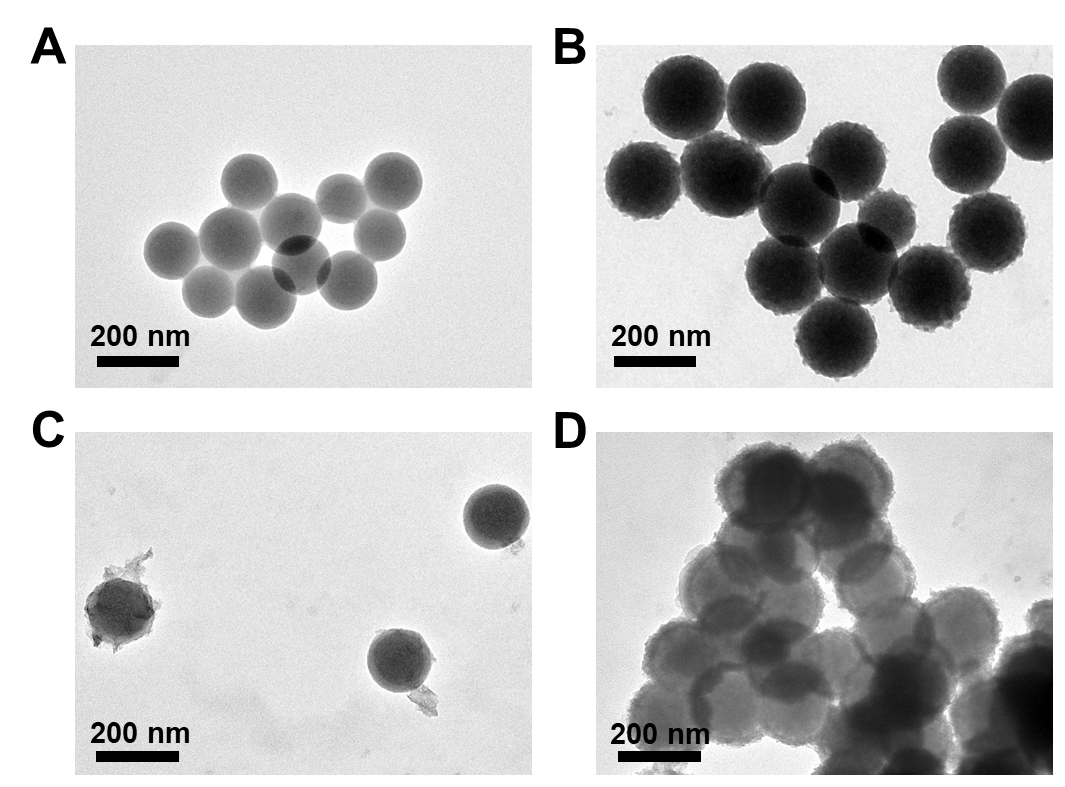
**

**FIGURE S1** TEM images of (A) SiO_2_, (B) SiO_2_@MnO_2_, (C) SiO_2_@PDA, and (D) SiO_2_@PDA@MnO_2_ nanoparticles.


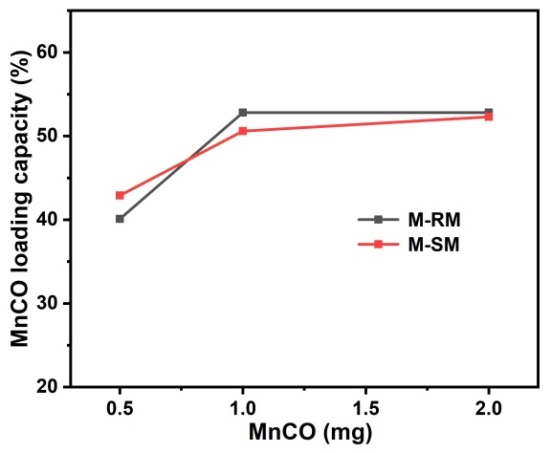


**FIGURE S2** MnCO loading efficiency in RM and SM after different amounts of MnCO were added.


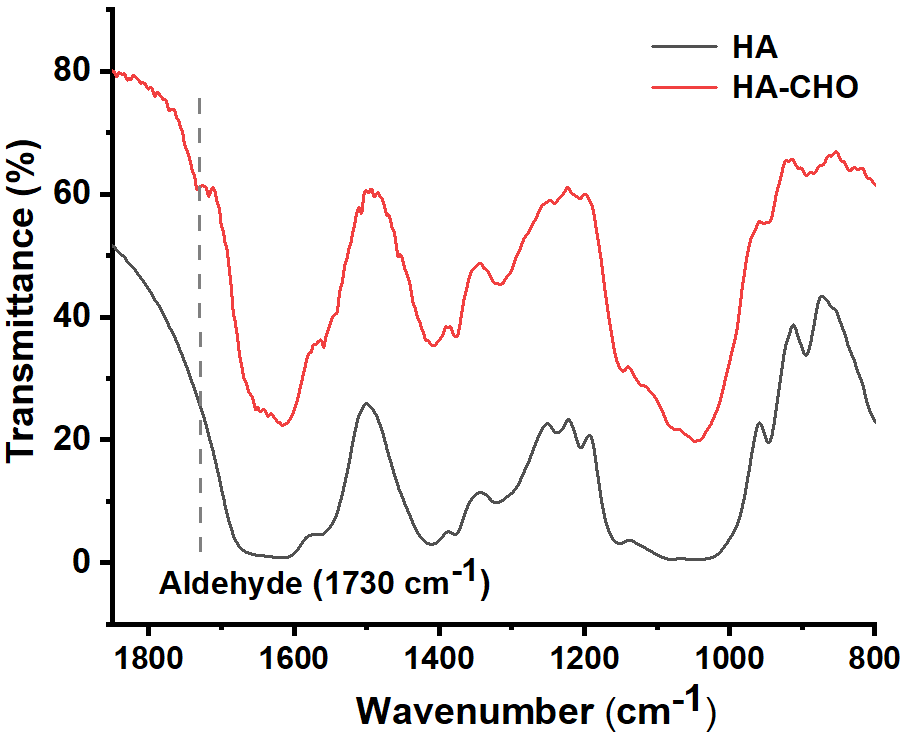


**FIGURE S3** FTIR spectra of HA and HA-CHO.


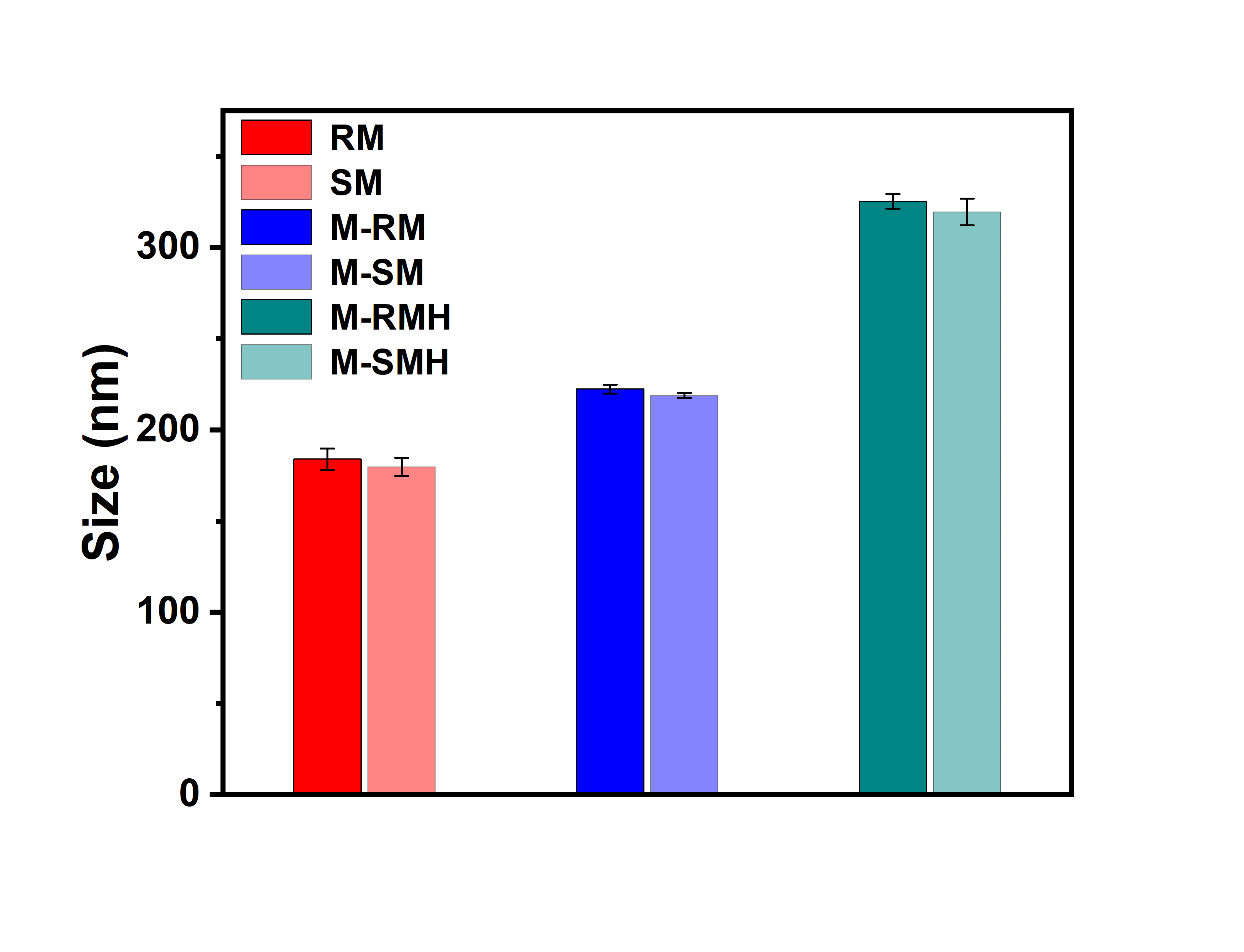


**FIGURE S4** Hydrodynamic sizes of RM, SM, M-RM, M-SM, M-RMH, and M-SMH (Mean ± SD, *n* = 3).


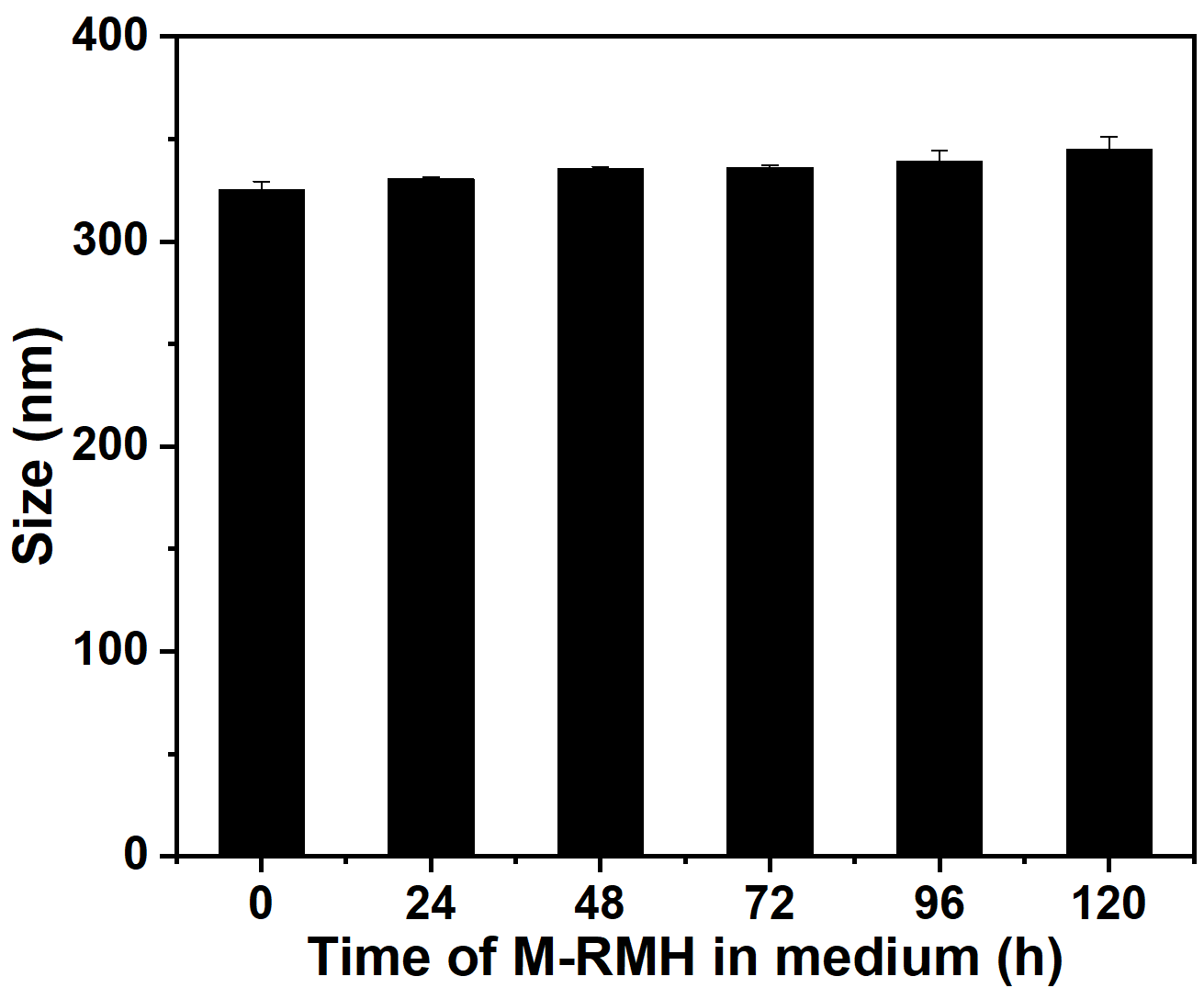


**FIGURE S5** Stability of M-RMH. Particle size by DLS of M-RMH incubated in 10% fetal bovine serum (Mean ± SD, *n* = 3).

**
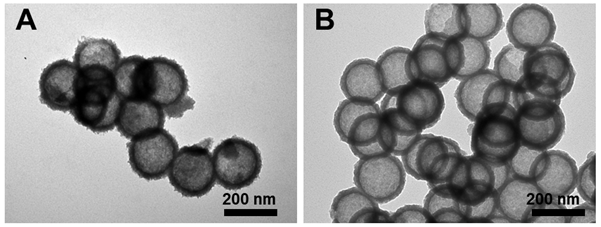
**

**FIGURE S6** TEM images of (A) M-RMH and (B) M-SMH nanoparticles.


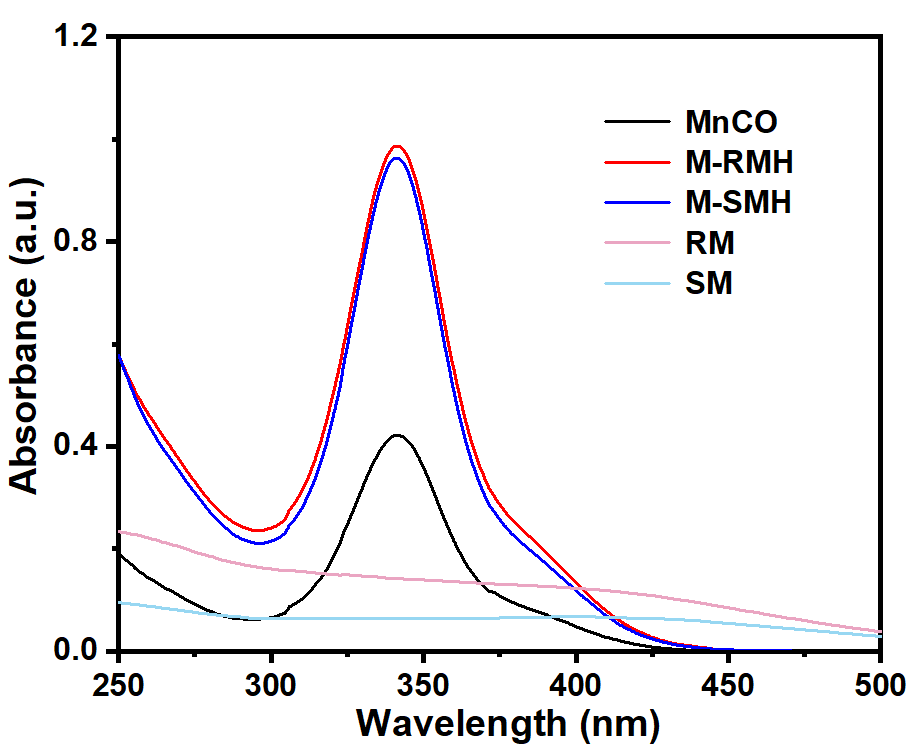


**FIGURE S7** UV-vis absorption spectrum of MnCO, RM, SM, M-RMH, and M-SMH.


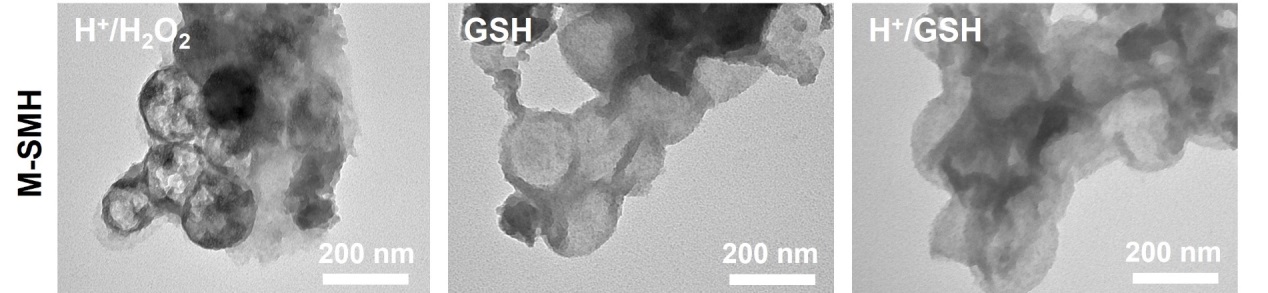


**FIGURE S8** TEM images of M-SMH after different treatments.


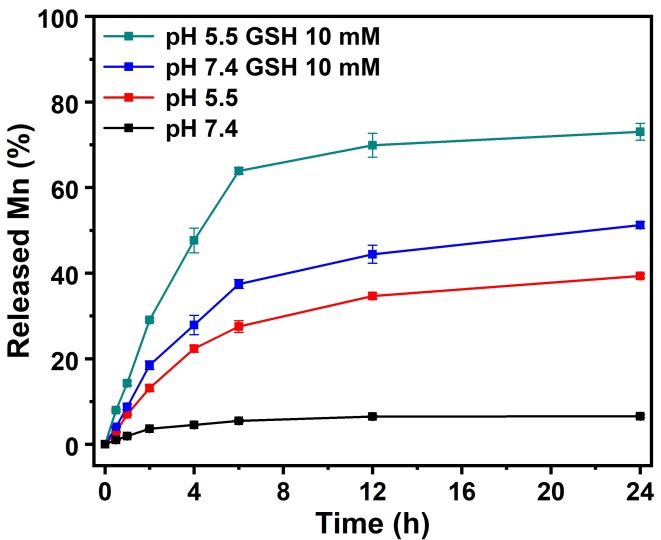


**FIGURE S9** Accumulated release profiles of Mn^2+^ from RMH in different conditions.


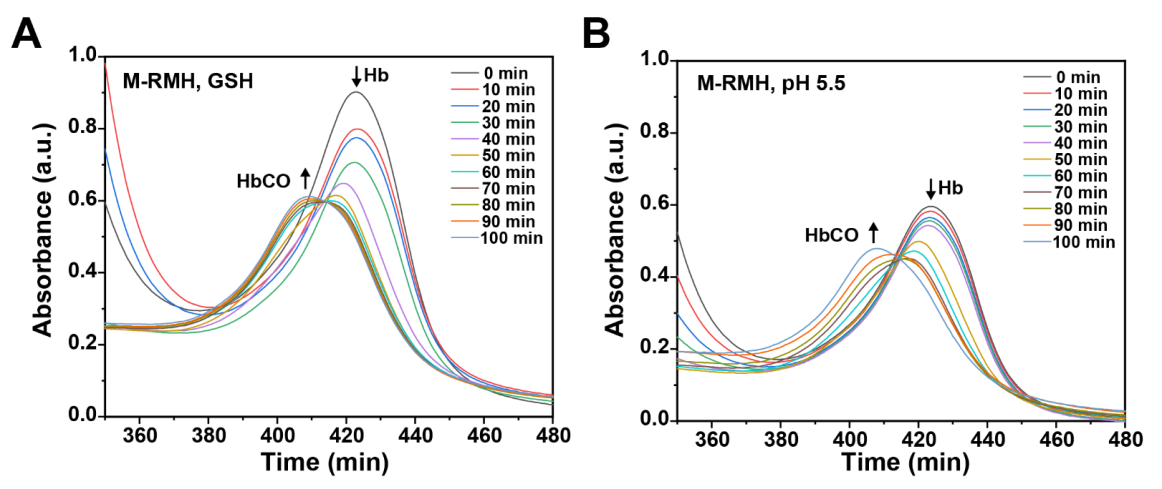


**FIGURE S10** UV-vis absorbance of HbCO indicating CO release from M-RMH in PBS containing 100 μM H_2_O_2_.


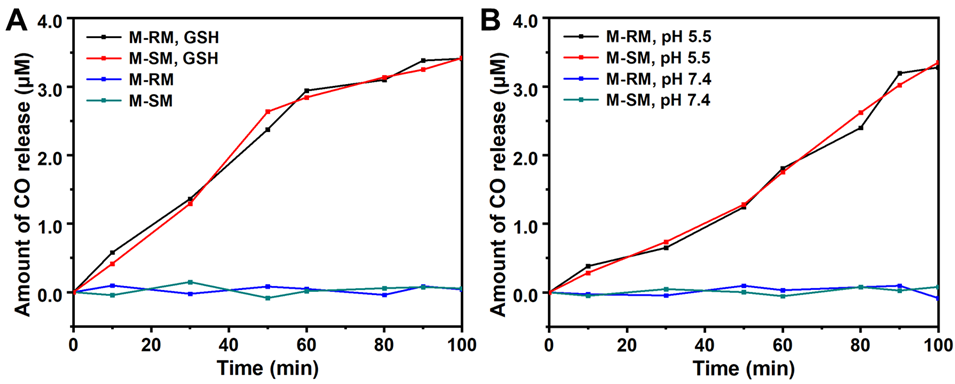


**FIGURE S11** CO release profiles from M-RM or M-SM in buffer solutions containing H_2_O_2_ with or without GSH (A), and with different pH values (B).


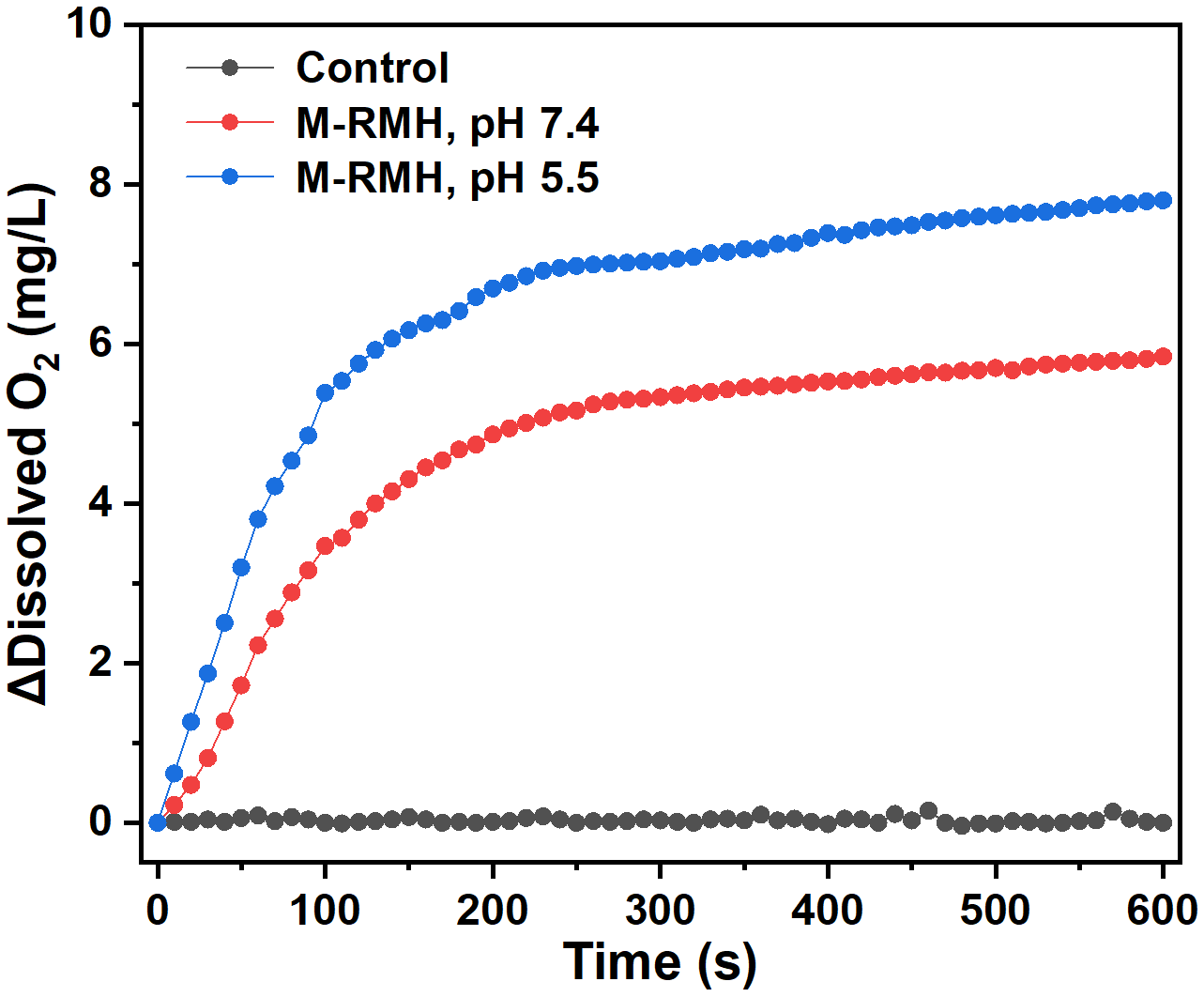


**FIGURE S12** Oxygen generation in 100 μм H_2_O_2_ in the presence of M-RMH with different pH values.


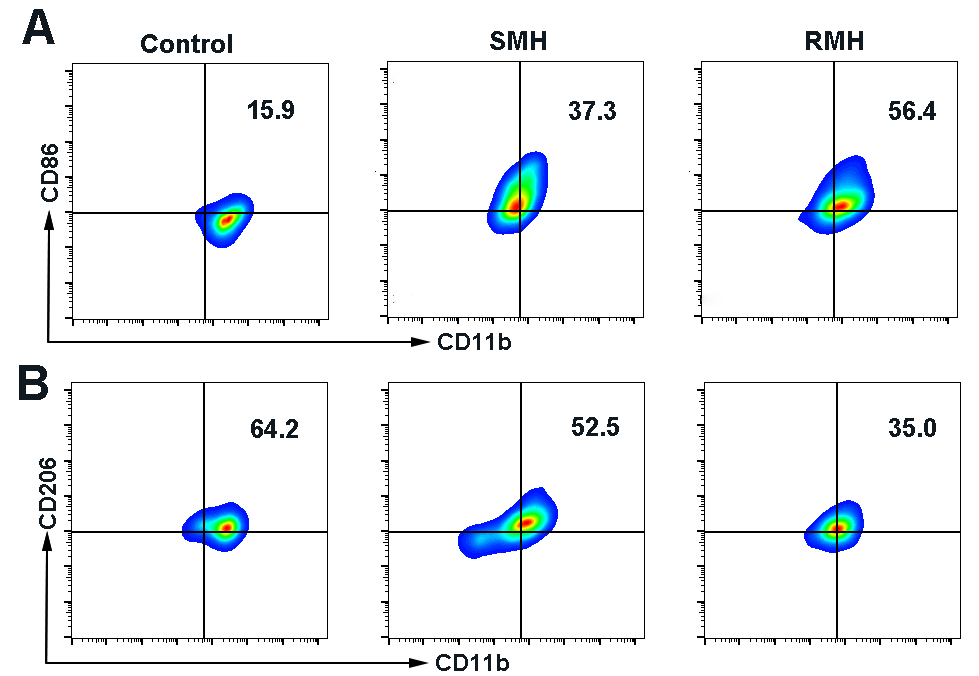


**FIGURE S13** Flow cytometric analysis of (A) CD86 (M1 macrophage marker) and (B) CD206 (M2 macrophage marker) expression in RAW264.7 cells after incubation with RMH and SMH.


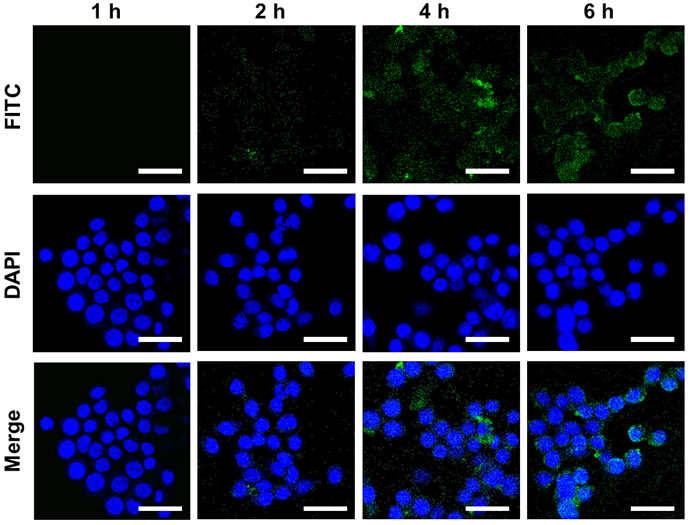


**FIGURE S14** CLSM images of RAW264.7 cells treated with M-RMH for different time. Scale bar: 25 μm.


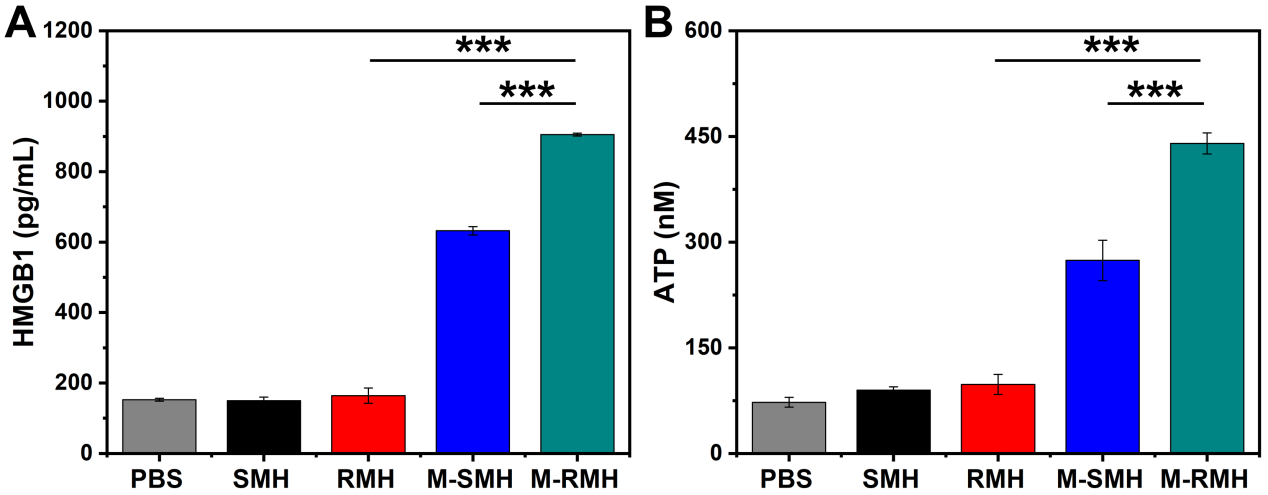


**FIGURE S15** Extracellular release of b) HMGB1 and c) ATP from 4T1 cells after different treatments. (Mean ± SD, *n* = 3). ****P* < 0.001, analyzed by one-way ANOVA with Tukey’s test.


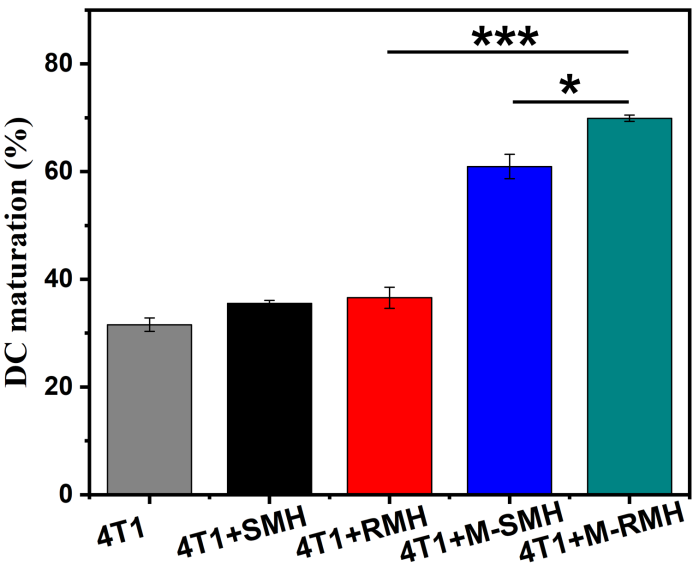


**FIGURE S16** Flow cytometry of CD80 and CD86 expression on BMDCs after different treatments. (Mean ± SD, *n* = 3). **P* < 0.05, ****P* < 0.001, analyzed by one-way ANOVA with Tukey’s test.


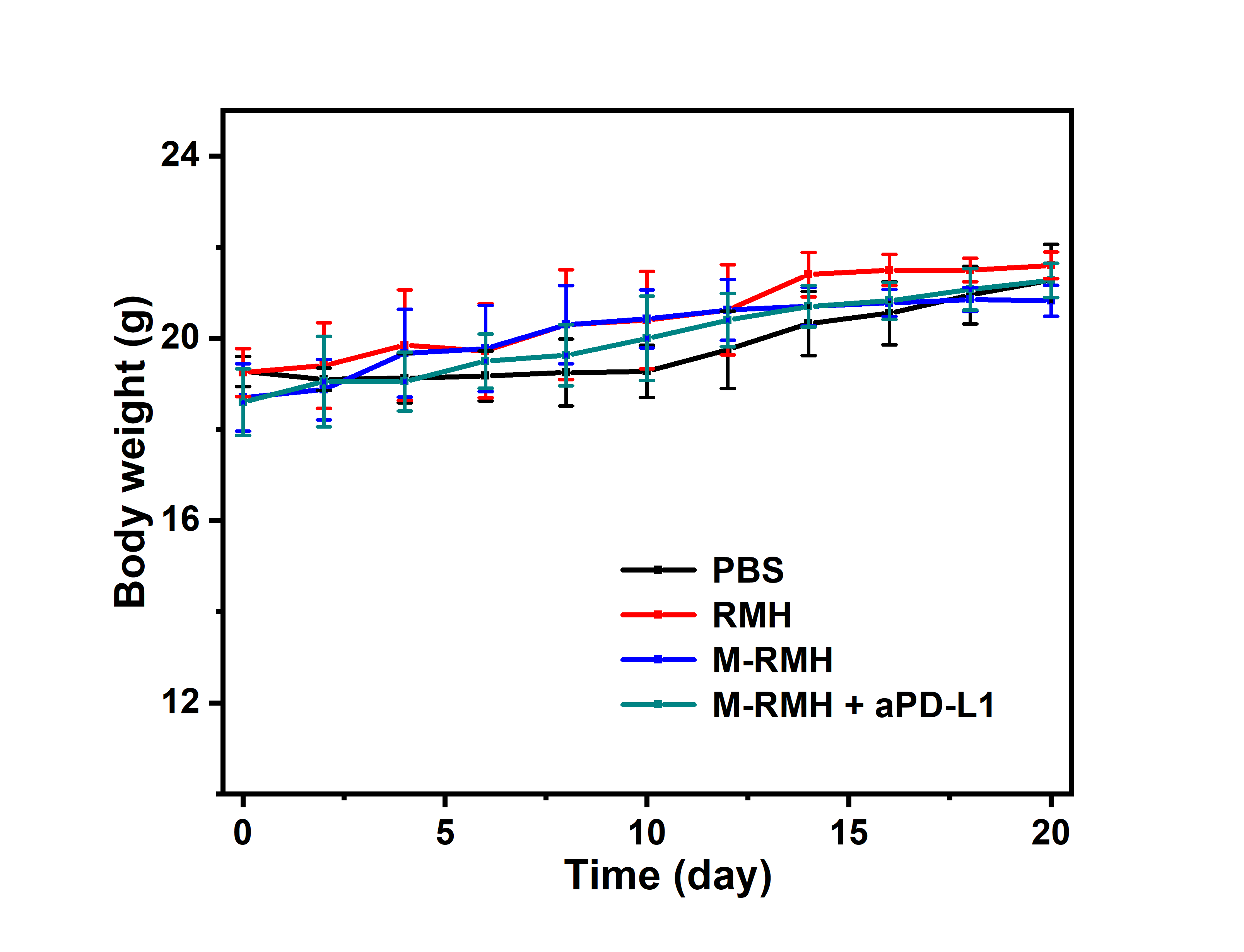


**FIGURE S17** Body weight evolution of tumor-bearing mice following different treatments (Mean ± SD, *n* = 4).

**
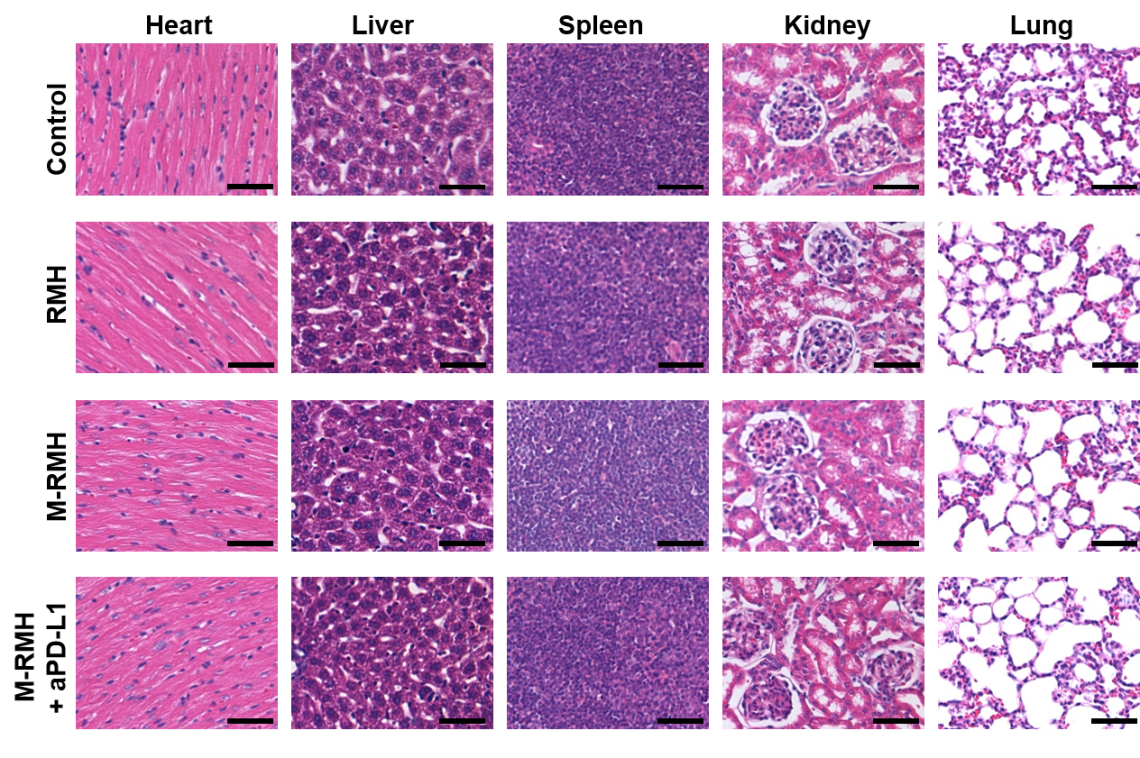
**

**FIGURE S18** H&E staining of major organs (heart, liver, spleen, kidney, and lung) in mice after different treatments. Scale bar: 50 μm.


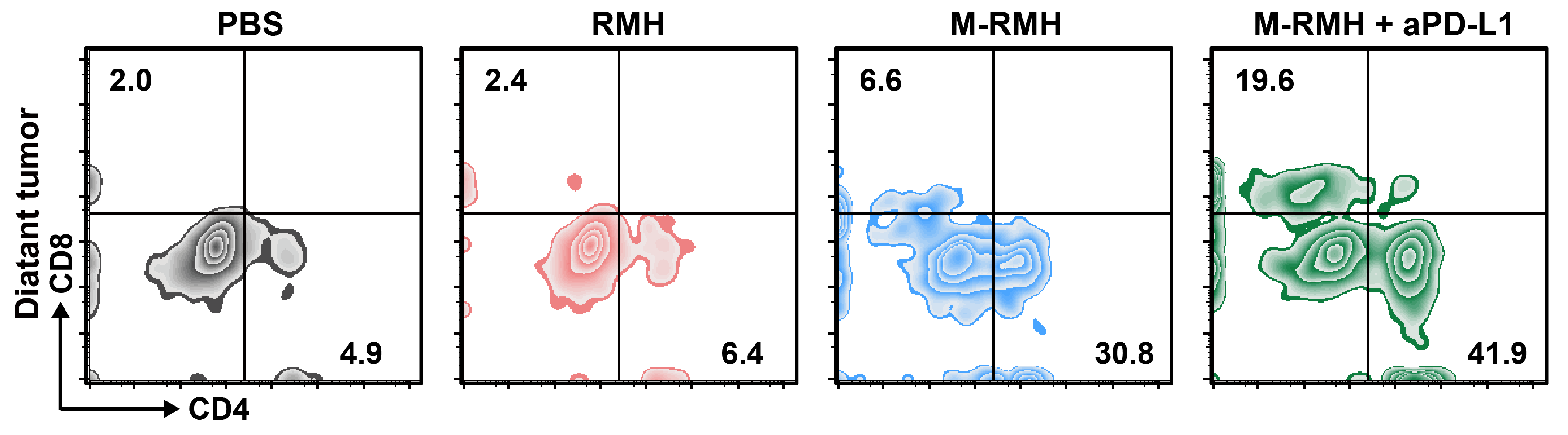


**FIGURE S19** Representative flow cytometry plots of CD4^+^ and CD8^+^ T cells in distant tumors after different treatments.


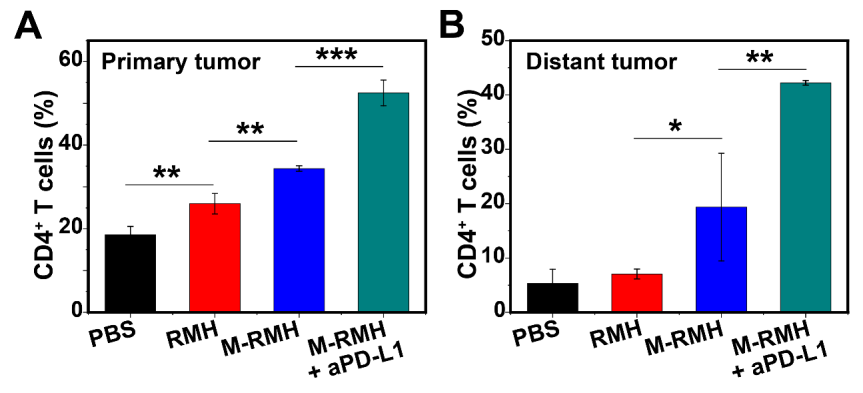


**FIGURE S20** Quantification results of CD4^+^ T cells in (A) primary tumors and (B) distant tumors of mice (Mean ± SD, *n* = 3). **P* < 0.05, ***P* < 0.01, ****P* < 0.001, analyzed by one-way ANOVA with Tukey’s test.


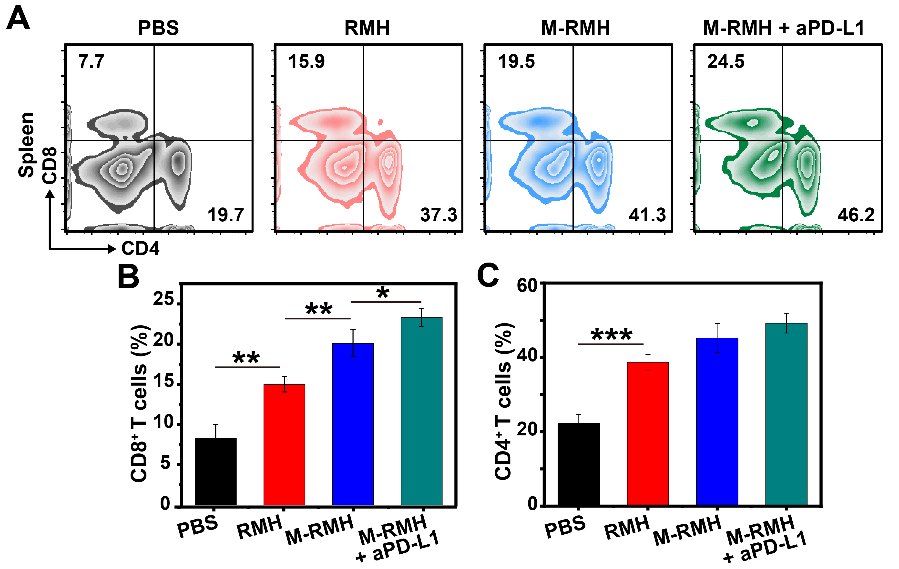


**FIGURE S21** (A) Representative flow cytometry plots of CD4^+^ T cells in spleens. Quantification results of (B) CD8^+^ T cells and (C) CD4^+^ T cells in spleens of mice after different treatments (Mean ± SD, *n* = 3). **P* < 0.05, ***P* < 0.01, ****P* < 0.001, analyzed by one-way ANOVA with Tukey’s test.


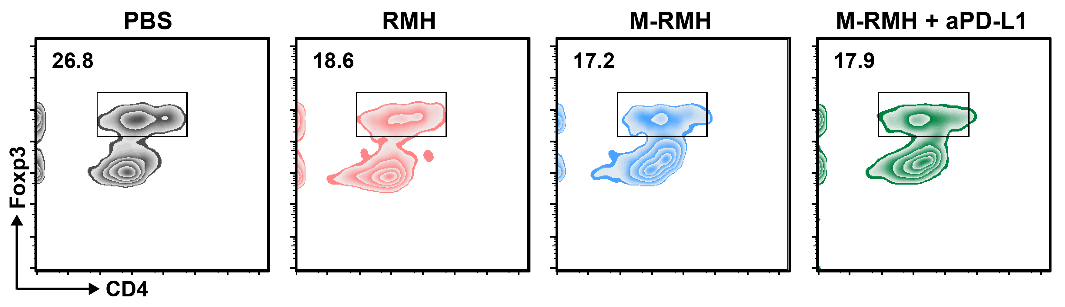


**FIGURE S22** Representative flow cytometry plots of Tregs (CD4^+^Foxp3^+^, gating on CD3^+^ T cells) in primary tumors after different treatments.


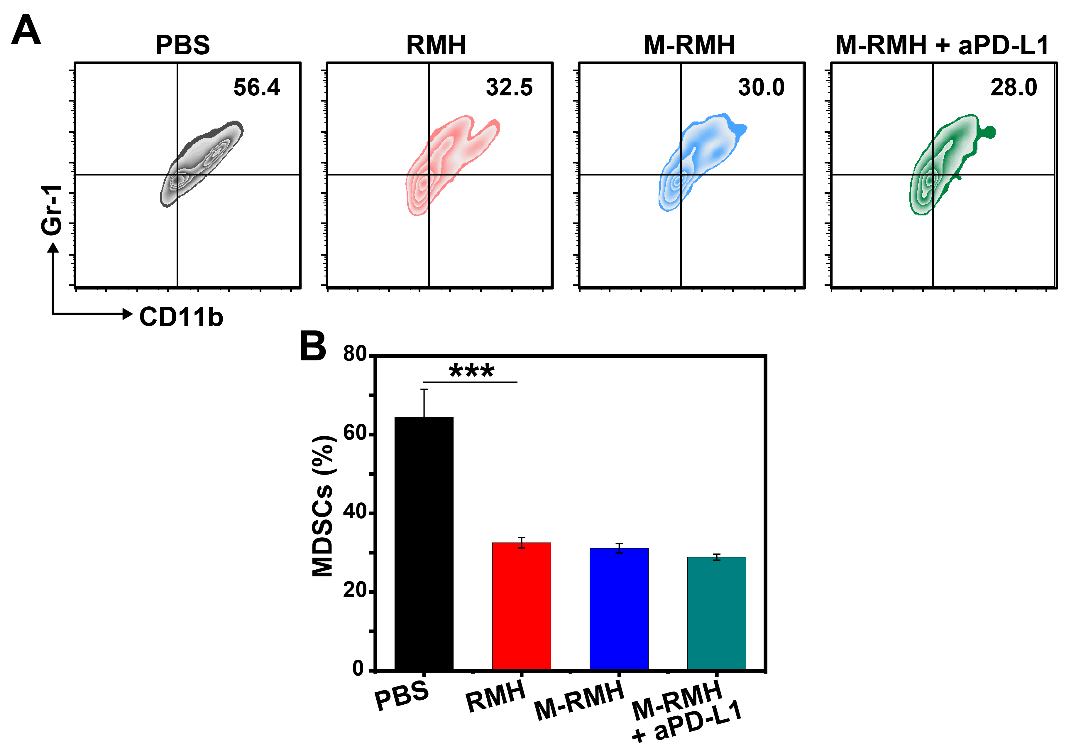


**FIGURE S23** (A) Representative flow cytometry plots and (B) quantitative analysis of MDSCs (CD45^+^CD11b^+^Gr-1^+^) in primary tumors after different treatments (Mean ± SD, *n* = 3). ****P* < 0.001, analyzed by one-way ANOVA with Tukey’s test.

**References**

1. [] G. Yang, L. Xu, Y. Chao, J. Xu, X. Sun, Y. Wu, R. Peng, Z. Liu, *Nat. Commun.* **2017**, *8*, 902-914. [↑](#endnote-ref-1)
2. [] Y. Zhang, L. Liu, L. Lin, J. Chen, H. Tian, X. Chen, A. Maruyama, *Acta Biomater.* **2018**, *65*, 349-362. [↑](#endnote-ref-2)
3. [] H. Zhao, N. D. Heindel, *Pharm. Res*. **1991**, *8*, 400-402. [↑](#endnote-ref-3)
4. [] D. Wu, X. Duan, Q. Guan, J. Liu, X. Yang, F. Zhang, P. Huang, J. Shen, X. Shuai, Z. Cao, *Adv. Funct. Mater.* **2019**, *29*, 1900095. [↑](#endnote-ref-4)
5. [] K. Inaba, M. Inaba, N. Romani, H. Aya, M. Deguchi, S. Ikehara, S. Muramatsu, R. M. Steinman, *J. Exp. Med.* **1992**, *176*, 1693-1702. [↑](#endnote-ref-5)
6. [] X. Zhao, K. Guo, K. Zhang, S. Duan, M. Chen, N. Zhao, F. J. Xu, *Adv. Mater.* **2022**, *34*, 2108263. [↑](#endnote-ref-6)
